# Metabolomic Fingerprinting in the Comprehensive Study of Liver Changes Associated with Onion Supplementation in Hypercholesterolemic Wistar Rats

**DOI:** 10.3390/ijms18020267

**Published:** 2017-01-28

**Authors:** Diana González-Peña, Danuta Dudzik, Antonia García, Begoña de Ancos, Coral Barbas, Concepción Sánchez-Moreno

**Affiliations:** 1 Institute of Food Science, Technology and Nutrition (ICTAN), Spanish National Research Council (CSIC), ES-28040 Madrid, Spain; diana.gonzalez@ictan.csic.es (D.G.-P.); ancos@ictan.csic.es (B.d.A.); 2 Center for Metabolomics and Bioanalysis (CEMBIO), Faculty of Pharmacy, San Pablo CEU University, Boadilla del Monte, ES-28668 Madrid, Spain; danuta.dudzik@ceu.es (D.D.); antogar@ceu.es (A.G.)

**Keywords:** functional food, hypercholesterolemia, mass spectrometry, LC-MS, CE-MS, GC-MS, metabolome, non-targeted metabolomics, non-alcoholic fatty liver disease (NAFLD)

## Abstract

The consumption of functional ingredients has been suggested to be a complementary tool for the prevention and management of liver disease. In this light, processed onion can be considered as a source of multiple bioactive compounds with hepatoprotective properties. The liver fingerprint of male Wistar rats (*n* = 24) fed with three experimental diets (control (C), high-cholesterol (HC), and high-cholesterol enriched with onion (HCO) diets) was obtained through a non-targeted, multiplatform metabolomics approach to produce broad metabolite coverage. LC-MS, CE-MS and GC-MS results were subjected to univariate and multivariate analyses, providing a list of significant metabolites. All data were merged in order to figure out the most relevant metabolites that were modified by the onion ingredient. Several relevant metabolic changes and related metabolic pathways were found to be impacted by both HC and HCO diet. The model highlighted several metabolites (such as hydroxybutyryl carnitine and palmitoyl carnitine) modified by the HCO diet. These findings could suggest potential impairments in the energy−lipid metabolism, perturbations in the tricarboxylic acid cycle (TCA) cycle and β-oxidation modulated by the onion supplementation in the core of hepatic dysfunction. Metabolomics shows to be a valuable tool to evaluate the effects of complementary dietetic approaches directed to hepatic damage amelioration or non-alcoholic fatty liver disease (NAFLD) prevention.

## 1. Introduction

Hypercholesterolemia is a recognized risk factor in the initial stages of hepatic damage, acting as an accelerator in the onset and worsening of non-alcoholic fatty liver disease (NAFLD) [1]. The initial stages of NAFLD may evolve toward a broad spectrum of liver abnormalities progressing from non-alcoholic steatohepatitis (NASH) to cirrhosis and hepatocellular carcinoma (HCC) [2]. These pathological conditions may confer additional risk to vascular dysfunction at very early stages [3].

The impact of the diet in health status is multifactorial, but stimulates an especially fast response in liver functions. As the liver is highly influenced by the feeding pattern, its functions are also amenable to change and regulate in response to modifications in the diet composition. Both pathological circumstances requiring clinical intervention as well as a good health status may profit from the consumption of functional ingredients. These functional ingredients may intercede in the regulation of the mechanisms of liver damage, offering new possibilities as complementary tools in the management of liver disease [4,5].

Nutritional and food research have rapidly evolved in the last decades with the development and application of new technologies. Many studies have pointed out onion as a source of multiple bioactive substances that are able to confer potential benefits on health and imbalances such as lipid alterations, inflammation and oxidative stress present in the initiation of most diseases [6,7]. The hepatoprotective effect of onion has been presented in diverse models of liver injury [8,9]. A recent study in experimental fed rats has revealed that onion consumption, as a part of a healthy diet, may be effective for some features in the management of NAFLD [10]. Moreover, it is of interest that the in vivo bioavailability and functionality of onion may be improved by the application of processing technologies such as high-pressure processing [11,12]. However, the in vivo evaluation of this onion ingredient from an integrative perspective is a complex compendium of research work where its possible preventive effects and/or the enhancement of pro-resolution pathway responses to hypercholesterolemia have to be considered.

The liver is a key metabolic organ that draws a great amount of attention in metabolomics with the endeavor to clarify the complexity of the liver metabolome [13]. Although the trend is to give priority to non-invasive or minimally invasive techniques, the direct analysis of tissue samples may provide fundamental information to understand the metabolic changes triggered by specific diseases. Nevertheless, an additional limitation, which should be taken into consideration when analyzing liver tissues is that derived from the methodological difficulties added in the pre-treatment, homogenization and metabolite extraction. As a result, the liver has attracted the largest number of non-targeted metabolomics studies [14], facing new challenges to interpret the interplay of pathways [15]. Furthermore, given the influence of diet, elucidating the interaction of specific foods or their components in the liver metabolic responses is especially important for the success of future approaches which aim to give an individualized dietary recommendation [4,16].

Therefore, the present study combines the analysis of three non-targeted metabolomic platfoms LC-MS, CE-MS and GC-MS to obtain the liver fingerprint of induced hypercholesterolemia in Wistar rats. Simultaneously, the extent to which processed onion may affect the liver regulation and prevent the induced metabolic imbalance will be assessed. These objectives rely on the emerging and promising field of foodomics in which the assessment of food benefits and risks can help not only to understand the pathophysiology of prevalent diseases such as NAFLD but also to identify novel metabolic biomarkers.

## 2. Results and Discussion

**Figure 1:**
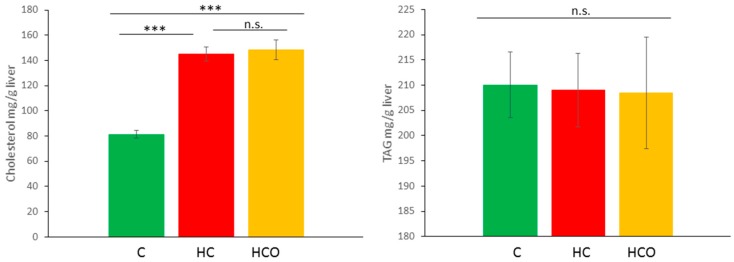
Liver cholesterol and triacylglycerides (TAG) concentration after seven weeks of experimental feeding. n.s., not significant; Control (C) diet, High-cholesterol (HC) diet, High-cholesterol enriched with onion (HCO) diet. *** *p* < 0.0001.

The pathological changes observed in the liver after seven weeks of high-cholesterol (HC) feeding corresponded with NAFLD symptomatology showing an evident hepatomegaly mainly due to lipid accumulation; altered biochemical parameters such as aspartate aminotransferase (AST), alanine aminotransferase (ALT) enzymes and modified activity of antioxidant enzymes glutathione peroxidase (GPx), catalase (CAT) and superoxide dismutase (SOD) (data not shown). The levels of total cholesterol in liver were higher in hypercholesterolemic rats (HC and HCO groups). Nevertheless, the measurement of total triacylglycerols (TAG) did not show statistical difference among the groups (Figure 1). Whereas an impaired hepatic function was clearly associated in the group fed the HC diet, the group fed the HCO diet evidenced a significant enhancement of the antioxidant defense system. The inclusion of the onion ingredient maintained liver CAT activity at the same level as the C group and induced the increase of GPx and SOD activities, although removal of cholesterol and TAG from the liver was not significantly improved (Figure 1). These changes, along with others related to the amelioration of the vascular status [17], need further exploration of the mechanism of action engaged in the hepatoprotection conferred by the consumption of processed onion in the course of hypercholesterolemia.

The natural antioxidant and anti-inflammatory potential of this onion ingredient has been previously demonstrated in vitro, indicating that it possess a strong antioxidant and free radical scavenging capacity [12]. Moreover, the same rats and experimental design was used to elucidate the differential plasma pattern induced by the HC diet and the influence of the onion ingredient [18]. This metabolic screening indicated specific metabolites and related metabolic pathways, which have encouraged the development of multiplatform metabolic fingerprinting of liver tissues. In addition, the present study has been motivated by a recent work that showed that onion supplementation ameliorated certain HC diet-related changes in liver oxylipins, which might be linked with a positive impact on the hepatic inflammatory process [19].

Therefore, three complementary non-targeted analytical platforms measuring different chemical nature of compounds (LC-MS, GC-MS and CE-MS) were successfully optimized and used to analyze the liver tissues. The results obtained by each technique are presented individually and, in a final section, analyzed together using multivariate analysis. The integration of all these results indicates the suitability of the techniques and offers a more comprehensive picture of the changes promoted by HC and HCO diets into the liver metabolome.

### 2.1. Liver Metabolic Fingerprinting by LC−MS

1169 and 490 features (ESI(+) and (–) ionization mode, respectively) were obtained after alignment, manual screening and integration correction of the whole set of samples fingerprinted by LC-MS. These data matrix were used for statistical evaluation. In total 618 features in ESI(+) mode and 369 in ESI(−) mode were found to be statistically significant. In order to select the most relevant outcomes a threshold of two-fold change in post hoc test for HC vs. C and HCO vs. C comparison and 1.5-fold change for HCO vs. HC was established before tentative identification.

**Figure 2:**
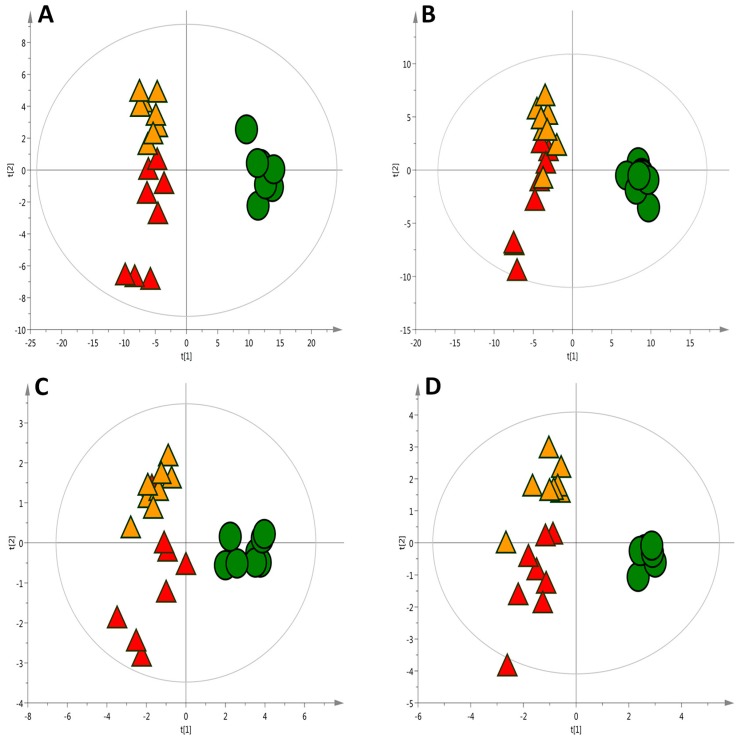
PLS-DA scatter score plots of liver metabolic profiles under seven week of experimental feeding. ● Control (C) diet, ▲ High-cholesterol (HC) diet, ▲ High-cholesterol enriched with onion (HCO) diet. (**A**) LC-MS ESI(+) (*R*^2^ = 0.80, *Q*^2^ = 0.68); (**B**) LC-MS ESI(−) (*R*^2^ = 0.73, *Q*^2^ = 67); (**C**) CE-MS (*R*^2^ = 0.72, *Q*^2^ = 0.55); (**D**) GC-MS (*R*^2^ = 0.79, *Q*^2^ = 0.71). *R*^2^ = coefficient for variance explained; *Q*^2^ = coefficient for variance predicted.

To estimate the predictive ability of the multivariate Partial Least Squared Discriminant Analysis (PLS-DA) models (Figure 2A,B), cross-validation based on the filtered data matrix were performed, resulting in 97% (ESI+) and 84% (ESI−) samples classified correctly.

The list of putatively identified compounds from univariate analysis is shown in Supplementary Table S1. This methodology revealed the most important changes in the HC group compared with C group, demonstrating profound impairments in the lipid metabolism as outlined by the tremendous changes in most metabolites detected, which belong to the family of glycerolipids, glycerophospholipids, fatty acyls, sphingolipids, steroids and steroid derivatives. The study highlighted the exorbitant increase in glycerophospholipids together with an unexpected decrease in some TAG. Diet induced hypercholesterolemic models, either composed of fats or cholesterol, are usually characterized by an increase in TAG [20,21]. However, the HC diet administered in the present study was composed of cholesterol and cholic acid. The inclusion of bile acids in the diet has been reported to affect the TAG homeostasis, being able to lower TAG. Watanabe et al. demonstrated the capability of cholic acid to lower both circulating and hepatic TAG levels in *KK-A*^y^ mice and proposed a suitable mechanism to explain this effect via a pathway involving farnesoid X receptor (FXR), short heterodimer partner (SHP), and sterol regulatory element binding protein-1c (SREBP-1c) [22]. In this sense, the present study also reveals an increase in hepatic diacylglycerols (DAG), which might compensate TAG levels and indicate an increased rate of conversion from AG to DAG and/or a diminished synthesis of TAG that could be favored with HC feeding in this animal model [20,21]. In addition, the condition of fasting previous to the animal sacrifice could have favored the TAG degradation by adipose triglyceride lipase (ATGL), facilitating the increase in total amounts of DAG [23]. The lipidomic profile found in the present study corroborated that cholesterol and its derivatives were increased by the HC diet, while the HCO diet did not ameliorate these concentrations in the liver (Table 1). However, the exact underlying mechanism that characterized the lipid hallmark of the liver in this specific model requires further investigation.

On the other hand, disturbances in carnitine and acylcarnitines homeostasis were also detected in liver tissues, which may correspond to alterations found in plasma [24]. Moreover, the influence of onion ingredient supplementation in the liver tissue has been notably marked in certain metabolites, whose assigned putative identification matched with metabolites in the carbohydrate class (e.g., glucopyranosyl-glucopyranosyl-glucose, glucose, lactose) and some glycosylated forms (e.g., sphingolipids). These changes reflect the mitigation of the reduced values in the HC group or increased values in the HCO group compared with the HC group (Table S1). Although some important fatty acids such as hexadecenoic acid, octadecatrienoic acid and arachidonic acid were decreased in the HC and HCO groups, an increase in other fatty acids and most phospholipids (PC/PE) and lysophospholipids (LPC) was found in the HCO group compared to both the C and HC groups. Potential modulating effects of the onion ingredient were also detected in tentatively identified sphingomyelins. In this sense, it is known that the expression of hepatic fatty acid elongase and desaturase is regulated by nutrients, hormones and nuclear receptor agonists [25]. Additionally, onion and quercetin and quercetin derivatives, which are the main flavonols in onion, are able to affect the fatty acid metabolism by modifying the activity of numerous enzymes such as lipoxygenase (LOX), cyclooxygenase (COX) and cytochrome P450 (CYP) [19,26,27,28]. They are also able to alter the expression profiles of several lipid metabolism-related genes [29]. As an example, a recent study in rats administered quercetin-3-*O*-β-d-glucuronide demonstrated the amelioration of free fatty acids in NAFLD [30]. However, these studies represent a part of the multiplicity of altering factors that make difficult the interpretation of changes in lipids in the present study. This complexity justifies the ambiguity of information concerning the lipid metabolism and metabolic regulation, which is also dependent on the animal model used and experimental conditions [31].

### 2.2. Liver Metabolic Fingerprinting by CE-MS

Extraction of metabolite features for CE-MS was accomplished by using the same tools as for LC-MS analysis. In this case, 175 features were initially aligned and further reduced to 129 features by manual data screening (isotopic pattern, peak shape evaluation, formation of multiple adducts, etc.). Multivariate unsupervised (PCA) and supervised (PLS-DA) modelling as well as univariate statistical analysis were performed. Figure 2C shows the corresponding score plot for the PLS-DA with a clear separation observed among the three groups. The model quality was satisfactory with a variance predicted *R*^2^ = 0.72 and explained *Q*^2^ = 0.55 showing a 94% of the excluded samples classified correctly by cross validation approach. The univariate analysis revealed 67 statistically significant features from which 33 were linked to metabolite identities. Supplementary Table S2 shows the preliminary list of potential metabolites obtained by univariate analysis, indicating the % of changes, along with the regulation and significance within each group.

Resulting from CE-MS analysis, the main classes of compounds detected were amino acids, organic acids, and purines and pyridine derivatives. Among these metabolites, carnitine and derivatives were specially affected by the HC diet showing a decrease in liver concentrations in accordance with previous results in plasma [24]. Additionally, a feature with a mass 384.12 recognized as hydroxybutyrylcarnitine as an attributed identity was marked as the most outstandingly affected metabolite in both the HC-fed (21-fold increase in concentration relative to the C group) and the HCO-fed groups (with a 4-fold change decrease compared with the HC group). This acyl carnitine has received greater attention in recent years, due to its possible association with insulin resistance and the development of diabetes [32,33,34].

A decreased level of glycine, serine, threonine and histidine was also observed in the liver of HC-fed rats compared with the C group, whereas the levels of asparagine and valine increased. All these amino acids showed a tendency to decrease with the supplementation of onion. Dipeptides were equally decreased by both the HC and HCO diets. In parallel to these changes, the polyamine formation would have been altered as indicated by the down-regulation of spermine, spermidine and *S*-adenosyl-methionine found in both the HC and HCO groups. In this sense, the regulation of amino acids and polyamines differs from that found in plasma, which showed an up-regulation produced by the consumption of the HC diet in most amino acids and polyamines detected [18,24]. These patterns would suggest an alteration in the arginine metabolism and amino acid transport across membranes motivated by the HC feeding [35].

Other interesting findings are related to the possible alteration of the methionine pathway due to the cholesterol and cholic acid overload and the increase of available choline in the liver of the HC-fed rats, which showed a tendency to decrease in the HCO group compared with the HC group. Choline is an important constituent and precursor of other biological compounds such as glycerophosphocholines or acetylcholine. This component influences several process but is especially important in liver, forming part of phosphatidylcholine where, for example, it is fundamental for the packaging and export of TAG [36].

### 2.3. Liver Metabolic Fingerprinting by GC-MS

The separation of the groups with a PLS-DA model, displayed in Figure 2D, was obtained with the whole set of metabolites aligned. The univariate analysis differentiated 33 metabolites which are listed in Table S3. These metabolites belong to the organic acids and derivatives, fatty acyls, nucleic acid related compounds and carbohydrates and carbohydrate conjugates. In coherency with the results found in CE-MS, some amino acids levels (see also Table S2) declined in both the HC and HCO groups. This technique allowed for the detection of metabolites involved in the TCA cycle, which showed an impairment in its regulation caused by the HC diet. Succinic acid, fumaric acid and malic acid among others were marked as metabolites potentially modulated by onion supplementation. Apparently, the consumption of onion enhanced the modification of metabolites incited by the HC diet. However, TCA impairments have been correlated with multiple diseases where oxidative stress plays a key role, and it has been suggested that an ineffective TCA cycle might be an adaptive response directed at diminishing reactive oxygen species (ROS) production and extend cellular longevity [37,38]. Thereby, the effect of the onion ingredient in this metabolic knot should be carefully examined and needs further study.

In addition, hydroxybutyric acids were significantly decreased in the HCO group compared with the C and HC groups, giving insight into the possible inhibition of the onion ingredient in the production of ketone bodies and the β-oxidation of fatty acids. 3-hydroxybutyrate is formed during ketogenesis from acetyl CoA of the hepatic mitochondria. Elevated 3-hydroxybutyrate has been previously reported in plasma of hypercholesterolemic Wistar rats, where it was suggested as biomarker [39].

Other relevant findings were associated with the significant changes in several methylated forms and *O*-phosphocolamine. It is of interest that whereas the concentration of most methylated fatty acids was lower in the HC group compared with the C group, the HCO group showed a generalized increase in their concentrations relative to the HC group. Therefore it could be hypothesized that the methylation of fatty acids was modified by the onion supplementation. However, to the best of our knowledge, the metabolic pathway by which methylated fatty acids may be affected in liver dysfunction remains to be elucidated.

With regards to *O*-phosphocolamine disposal, it is known to have a direct implication in membrane stability due to its role in the synthesis and its release by the breakdown of phospholipids in tissues. Therefore, the significant increase in the HC group and decrease in the HCO group compared to the HC group add insights into the likely effects of onion consumption in the induced lipid hallmark in the development of hypercholesterolemia.

### 2.4. Global Metabolic Interpretation

It is noteworthy that integration of complementary data by different techniques may allow the generation of a broader metabolic mapping and therefore a deeper interpretation. Whereas LC-MS enables the detection of a wide range of low- to medium-polarity metabolites, including non- and semi-polar and non-volatile compounds that cannot be analyzed by GC-MS, the analytical target of CE-MS is the separation of small, water-soluble ions, where amino acids are the main class of metabolites detected. Complementary, GC-MS is a well-suited technique that includes low-polarity volatile metabolites of lipids and esters, and high-polarity metabolites of amino acids and organic acids converted into volatile derivatives.

**Figure 3:**
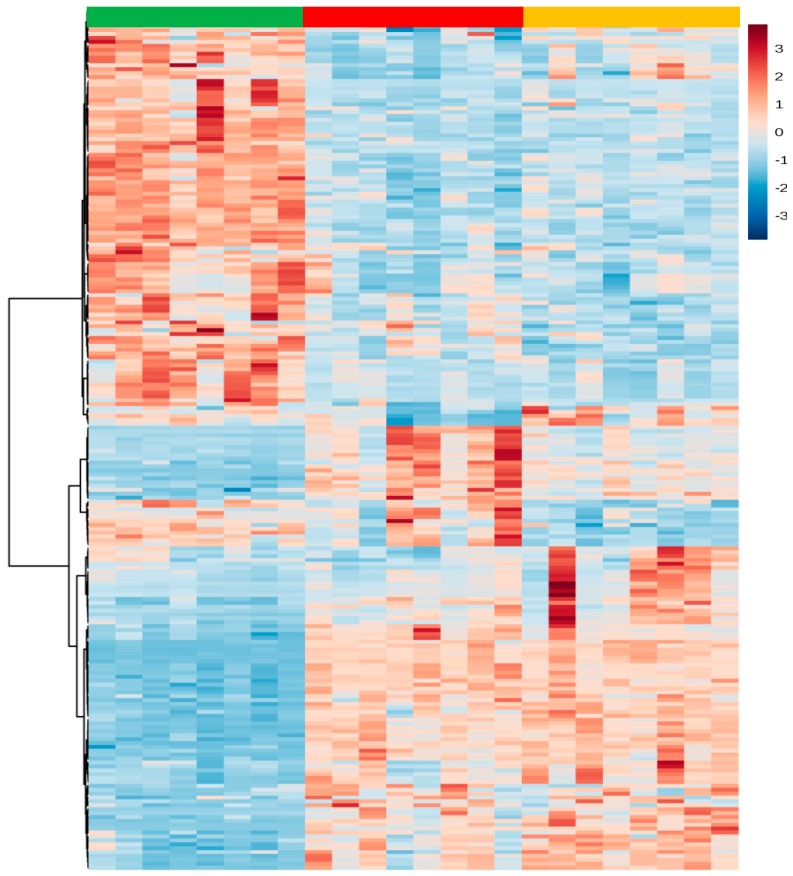
Heatmap of significant metabolites detected in liver tissues modified by C, HC and HCO diets. The color spectrum ranging from red to blue represents the range of high to low signal intensities, respectively, for each metabolite. ■ Control (C) diet, ■ High-cholesterol (HC) diet, ■ High-cholesterol enriched with onion (HCO) diet.

The differences in the regulation of metabolites within each experimental group in this study is evidenced by the magnitude of the changes represented in the heatmap (Figure 3), which shows an unique signature and characteristics metabolic pattern for the C, HC and HCO groups.

**Figure 4:**
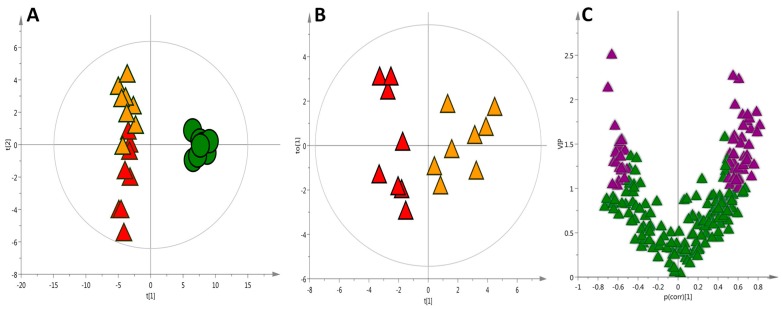
(**A**) PLS-DA scatter score plot of liver tissue after seven weeks of experimental feeding, *Q*^2^ = 0.97 and *R*^2^ = 0.97 [● Control (C), ▲ High-cholesterol (HC), ▲ High-cholesterol enriched with onion (HCO) group]; (**B**) OPLS scores plot visualizing the separation of the ▲ HC group and ▲ HCO group (*Q*^2^ = 0.82 and *R*^2^ = 0.50); (**C**) In order to identify metabolites with the highest influence on the group separation, variable selection using a combination of Variable Influence on the Projection (VIP) and p(corr) was performed. Variable selection using VIP > 1.0 and p(corr)| > 0.5 cutoff was applied (marked on purple ▲).

Likewise, the building of statistical models with more relevant information generates plots with better separation and predictive capability. This can be seen by the comparison of the quality (*Q*^2^ = 0.97 and *R*^2^ = 0.97) in the PLS-DA plot shown in Figure 4A (based on the complex data matrix of all putatively identified metabolites) with those created by each technique individually. The validation of this model resulted in 95% of the samples correctly classified. The merged data improved the separation of the HC and HCO groups, as presented in the Orthogonal Partial Least Squares Discriminant Analysis (OPLS-DA) projection (Figure 4B). Variable Influence on the Projection (VIP) ranks the original variables, according to their individual contribution to the model together with complementary p(corr). The correlation coefficient combined with VIP (VIP-p(corr) analysis) allowed the selection of the most relevant metabolites for the separation of the HC and HCO groups (Figure 4C; Table 1).

As seen in these results, the chemical composition of metabolites detected in liver and the concentration range vary largely. No single analytical platform could target all metabolites present in one sample. Therefore, each platform contributed with a differential type of metabolites intended to complete the metabolite coverage of the matrix contemplated for the application of multivariate analysis.

To the best of our knowledge, the metabolomics studies performed to date concerning the effects of onion supplementation may be split into two blocks: the first and more extensive block concerns the detection of metabolites related to onion consumption [40,41,42,43,44] and the second relates to the detection of changes induced by onion consumption in markers associated to certain health or disease status. Within these studies, the hepatoprotective effects of onion have been subjected to little analysis, mainly focused on known biomarkers that were addressed by target approaches [45,46,47]. Nevertheless, a global image of the modifications caused by consumption of onion in the metabolome by non-targeted approaches was not obtained until a previous study by our group [18]. The plasma fingerprint of hypercholesterolemic rats was obtained and is complemented by this current study, which focusses for the first time on the effects of an onion ingredient in the liver metabolome.

Therefore, the present study may help to generate new hypothesis about the preventive effect of onion consumption by indicating potential pathways of interest to be addressed in future research. In this sense, considering the identities assigned to metabolites, which have significant influence for the separation of the groups in the model, the study of the modulation exerted by onion in the energy-lipid metabolism is emphasized. This may be addressed by, for example, targeting the carnitine and its derivatives together with the perturbations in the lipid hepatic liver composition that accompanies the altered lipid metabolism. Additionally, the alterations in the regulation of enzymes and reactions involved in the TCA cycle, the changes stimulated in the bile acid synthesis and the modifications in the methylation mechanisms together with the rates of methylation are also suggested as interesting metabolic routes likely modulated by onion supplementation that should be considered in the course of hepatic dysfunction.

## 3. Materials and Methods

### 3.1. Experimental Design and Diets

The present study was approved by the Spanish Ministry of Science and Innovation Advisory Committee (Project AGL2010-15910 (subprogram ALI)) and by the Ethics Committee of the Complutense University of Madrid (Spain). All experiments were performed in compliance with the Directive 2010/63/UE regarding the protection of animals used for scientific purposes.

24 male Wistar rats (body weight ~245 ± 5 g) were obtained from Harlan Laboratories Models (Harlan, SL, Barcelona, Spain). The acclimatization of the animals was held under controlled conditions of light (12 h light/dark cycle) and temperature (22 ± 1 °C) for three days. Afterwards, they were randomly divided into three groups, housed in individual metabolic cages and adapted to the new type of diet (AIN-93M diet) [48] prior the beginning of the experiment.

The experimental feeding period lasted for 7 weeks, where three experimental semi-synthetic diets corresponding to the following classification, were facilitated: control (C) diet, a homogeneous mixture of 100% rodent diet; high-cholesterol (HC) diet, control diet with 2% cholesterol and 0.5% cholic acid, substituting an equal amount of maize starch; and high-cholesterol enriched with onion (HCO) diet, identical to the HC diet, but with 10% onion powder, balancing the dietary fibre with cellulose powder. A detailed description about the diets composition and the onion ingredient (including a brief explanation about the preparation) can be found in the Supplementary material (Tables S4 and S5).

During the experiment, food and water were provided *ad libitum*, food consumption was recorded daily and body weight weekly. At the end of the experiment, animals in fasting conditions were anaesthetized and euthanized by extracting blood by cardiac puncture. Blood was taken from the heart and organs were immediately extracted.

### 3.2. Liver Sampling and Homogenate Preparation

Collection of livers was conducted in aseptic conditions just after exsanguination. Livers were weighed and dissected into parts that were individually frozen in liquid nitrogen and immediately stored at −80°C until analysis. The liver homogenates were prepared with representative sections of tissue from the central lobe of liver. In brief, ~50 mg of tissue were weighed and homogenized in cold methanol:water (1:1) at a weight to volume ratio of 1:10. Tissue disruption and metabolite extraction were carried out with a TissueLyserLT bead-mill homogenizer (QIAGEN, Hilden, Germany), using 3 mm (mean diameter) glass beads, vibrating at 50 Hz for 5 min. Four repeated cycles with a 1 min break during which samples and TissueLyser adapter were cooled on ice.

Each homogenized sample was split into three aliquots, one of each was used for each of the three analytical techniques performed immediately after the extraction. The volumes used for metabolite extraction of each analysis were 100, 100 and 150 µL, for LC-MS, CE-MS and GC-MS, respectively. The method for tissue homogenization was adapted from [49].

### 3.3. Metabolite Extraction

#### 3.3.1. LC-MS Analysis

Homogenate (100 µL) was reconstituted in 100 µL of methanol and vortex-mixed for 15 min. Subsequently, 100 µL of methyl *tert*-butyl ether (MTBE) was added and vortex-mixed for 1 h at room temperature. Samples were centrifuged for 20 min at 4000× *g* at 20 °C. 100 µL of supernatant was used for LC-MS analysis.

#### 3.3.2. CE-MS Analysis

Homogenate (100 μL) was vortex mixed with 100 μL of 0.2 M formic acid, centrifuged (16,000× *g* 10 min, 4 °C) and transferred to a centrifree ultracentrifugation device (Millipore Ireland Ltd., Cork, Ireland) with 30 kDa protein cutoff filter for deprotenization through centrifugation (2000× *g*, 70 min, 4 °C). The filtrate was then transferred to the chromacol vial, dried using a SpeedVac Concentrator (Thermo Fisher Scientific, Waltham, MA, USA), and resuspended in 100 μL of 0.1 M formic acid with 0.2 mM methionine sulfone (IS) before CE-MS analysis.

#### 3.3.3. GC-MS Analysis

Homogenate (150 μL) protein was precipitated with cold methanol (300 μL) containing 10 μL/mL of pentadecanoic acid (IS) and separated by centrifugation (16,000× *g*, 15 min, 4 °C). Resulting supernatant (300 μL) was transferred to GC vial with insert and then evaporated to dryness (SpeedVac Concentrator, Thermo Fisher Scientific, Waltham, MA, USA). Then, 20 μL of *O*-methoxyamine hydrochloride in pyridine (15 mg/mL) was added to each GC vial, and mixture was vigorously vortex-mixed and ultrasonicate. Methoxymation was carried out in darkness, at room temperature for 16 h. *N*,*O*-Bis(trimethylsilyl)trifluoroacetamide (BSTFA) with 1% trimethylchlorosilane (TMCS) (20 μL) was then added as catalyst. For silylation process samples were heated in an oven for 1 h at 70 °C. Finally, 100 μL of heptane containing 10 ppm of C18:0 methyl ester (IS) was added to each GC vial and vortex-mixed before GC analysis.

#### 3.3.4. Randomization

All samples prepared were randomized before homogenization, metabolite extraction and for the corresponding analytical run.

### 3.4. QC Sample Preparation

Quality control (QC) samples were prepared by pooling equal volumes of liver tissue homogenate from each sample. Six QC samples were independently prepared for each technique following the same procedure as applied for the experimental samples. QC samples were analyzed throughout the run to provide a measurement of the system’s stability, performance and the reproducibility of the sample treatment procedure.

### 3.5. Metabolic Fingerprinting

#### 3.5.1. Liquid Chromatography-Quadrupole Time of Flight-Mass Spectrometry (LC-QTOF/MS Analysis)

A HPLC system (1200 series, Agilent Technologies, Waldbronn, Germany), equipped with a degasser, two binary pumps, and a thermostated autosampler coupled with Q-TOF LC-MS (6520) system (Agilent Technologies, Waldbronn, Germany), was used in the ESI(+) and ESI(−) mode to increase the number of detected metabolite ions as previously described [49].

Briefly, 5 μL of extracted liver samples was injected into a thermostated (60 °C) Agilent Poroshell 120 EC-C8 column (150 × 2.1 mm, 2.7 μm; Agilent Technologies, CA, USA) with a guard column Ascentis^®^ Express C8 (5 × 2.1 mm, 2.7 μm; Supelco, Bellefonte, PA, USA). The flow rate was 0.5 mL/min with solvent A (5 mM ammonium formate in MilliQ^®^ water, Molsheim, France), and solvent B (5 mM ammonium formate in methanol and 15% of isopropanol) for analysis in positive ionization mode and solvent A (MilliQ^®^ water with 0.1% formic acid), and solvent B (methanol with 0.1% formic acid and 15% of isopropanol) for analysis in negative ionization mode. Initial conditions at time 0 were 82% B, increasing to 96% B in 30 min. This was then held until 38 min. The gradient then increased to 100% B by 38.5 min and held until 40.5 min. The starting condition was returned to by 42 min, followed by an 8 min re-equilibration time, taking the total run time to 50 min. Capillary voltage was set to 3.5 kV for positive and 4.5 kV for negative ionization mode; the drying gas flow rate was 12 L/min at 250 °C and gas nebulizer at 52 psi; fragmentor voltage was 175 V for positive and 125 V for negative ionization mode; skimmer and octopole radio frequency voltage (OCT RF Vpp) were set to 65 and 750 V, respectively. Data were collected in the centroid mode at a scan rate of 1.2 spectrum per second. Mass spectrometry detection was performed in both positive and negative ESI mode in full scan from 100 to 1200 *m*/*z*. The reference mass ions used were 121.050873, 922.009798 (positive ion mode) and 119.036320, 966.000725 (negative ion mode). These masses were continuously infused into the system to allow constant mass correction. Samples were analyzed in separate runs (positive and negative ionization modes), in a randomized order.

#### 3.5.2. Capillary Electrophoresis-Time of Flight-Mass Spectrometry (CE-TOF/MS Analysis)

An Agilent 7100 (CE) system, coupled to a TOF Mass Spectrometer (6224 Agilent), was used for sample analysis. In brief, a fused-silica capillary (Agilent Technologies; total length, 96 cm; i.d., 50 μm) was pre-conditioned with 1 M NaOH for 30 min, followed by MilliQ^®^ water and background electrolyte-BGE (0.8 M formic acid in 10% methanol) for 30 min. Before each analysis, the capillary was flushed for 5 min (950 mbar pressure) with BGE. The MS was operated in positive polarity, with a full scan from 80 to 1000 *m*/*z* at a rate of 1.4 scan/s. Drying gas was set to 10 L/min, nebulizer to 10 psi, voltage to 3.5 kV, fragmentor to 125 V, gas temperature to 200 °C and skimmer to 65 V. The sheath liquid composition was methanol/water (1/1, *v*/*v*), containing 1.0 mmol/L formic acid with two reference masses (121.050873-purine (C_5_H_4_N_4_) and 922.009798-HP-921 (C_18_H_18_O_6_N_3_P_3_F_24_)), which allows for correction and provides more accurate mass determination. Flow rate was 0.6 mL/min and split was set to 1/100. Samples were injected at 50 mbar for 50 s. After each injection, along with the samples, BGE was co-injected for 10 s at 100 mbar pressure to improve repeatability. Separations were performed at a pressure of 25 mbar and a voltage of +30 kV; current under these conditions was 100 μA.

#### 3.5.3. Gas Chromatography-Quadrupole-Mass Spectrometry (GC-Q/MS Analysis)

A GC system (Agilent Technologies 7890A), equipped with an autosampler (Agilent 7693) and interfaced to an inert mass spectrometer with triple-Axis detector (5975C, Agilent), was used for liver tissue fingerprinting. Briefly, 2 μL of the derivatized sample was injected in a GC column DB5-MS (30 m length, 0.25 mm, 0.25 μm film 95% dimethyl/5% diphenylpolysiloxane) coupled to a pre-column (10 mJ&W integrated with Agilent 122-5532G). The injector port was held at 250 °C, and the helium carrier gas flow rate was set at 1.0 mL/min. The split ratio was 1:10. The temperature gradient was programmed as follows: the initial oven temperature was set to 60 °C (held for 1 min), increased to 325 °C at a rate of 10 °C/min; the system was allowed to cool down for 10 min before the next injection. The detector transfer line, the filament source and the quadrupole temperature were set to 280, 230 and 150 °C, respectively. MS detection was performed in electron impact (EI) mode at −70 eV. The mass spectrometer was operated in scan mode over a mass range of 50–600 *m*/*z* at a rate of 2.7 scan/s.

#### 3.5.4. Data Treatment

Raw data acquired were processed to provide structured data in an appropriate format for data analysis. The data collected by LC-MS and CE-MS were cleaned of background noises and unrelated ions recursive analysis in MassHunter Profinder (B.06.00, Agilent Technologies) software. Feature extraction is the reduction of acquired data size and complexity through the removal of redundant and non-specific information by identifying the important variables (features) associated with the data. Molecular feature extraction (MFE) performs chromatographic deconvolution to find the features in the analyzed samples. The features are aligned across all of the selected sample files using mass and retention time. Recursive Feature Extraction first performs MFE and then uses the MFE results, feature mass and retention time, to perform a targeted feature extraction referred to as Find by Ion (FbI). Find by Ion uses the median mass, median retention time, and composite spectrum calculated from the aligned features to improve the reliability in finding the features in the data.

GC-MS data, peak detection and spectra processing algorithms were applied using the Agilent MSD ChemStation Software (G1701EA E.02.00.493, Agilent). Quality of the chromatograms acquired by total ion chromatography (TIC) for analyzed samples, QC samples, blanks and internal standard peak were carefully examined to overview of the overall quality of analytical performance. Automated Mass Spectrometry Deconvolution and Identification System (AMDIS) version 2.69 software from NIST (U.S. National Institute of Standards and Technology, Gaithersburg, MD, USA) was used for automatic mass spectral deconvolution to identify co-eluted compounds according to their retention indexes (RI) and retention times (RT). Retention times from analysis of fatty acid methyl ester standard solution was used to create a calibration data file for further adjustment of RT in samples. The accuracy improvement was based on the determination of the Kovats RI. Retention index value, contained in Fiehn RTL (Retention Time Locked) library was compared to the experimental RI value in order to assign a match score between the experimental and the theoretical spectra. Compounds were identified by comparing their mass fragmentation patterns with target metabolite Fiehn GC-MS Metabolomics RTL library (G1676AA, Agilent), the in-house CEMBIO-library and the NIST mass spectra library 2.0, using the ChemStation software and native PBM (Probability-Based Matching) algorithm (G1701EA E.02.00.493, Agilent). Alignment of drift (by retention time and mass) and data filtering were performed with the Mass Profiler Professional B.12.1 (Agilent) software. Data matrix was normalized according to internal standard pentadecanoic acid intensity.

Before statistical analysis, filtration of data matrix by samples frequency was applied. Variation of the compound concentrations in QC samples expressed as relative standard deviation (%RSD) was also calculated. A threshold of 20% for LC-MS and CE-MS and 30% for GC-MS was set for the RSD values of metabolites in the QC samples.

#### 3.5.5. Statistical Analysis

Data normality was verified by evaluation of the Kolmogorow−Smirnov−Lillefors and Shapiro−Wilk tests and variance ratio by the Levene’s test. Differences among experimental groups were tested by using either the ANOVA or the Kruskal−Wallis tests according to the distribution, normal or not, of the variables with post hoc Benjamini-Hochberg (FDR, false discovery rate) and Bonferroni test, respectively, for multiple comparisons. The levels of statistical significance were set at 95% level (*p* < 0.05). Statistical analyses were performed using Matlab R2015 (Mathworks) software. MetaboAnalyst data annotation tool was used for testing the relationships between variables [50]. Multivariate (unsupervised and supervised) analysis as well as other multivariate calculation and plots was performed by using SIMCA−P + 14.0 (Umetrics, Umeå, Sweden).

#### 3.5.6. Model Validation

PLS-DA models that were obtained according to multivariate calculations were validated by cross-validation tool [51]. Validation was performed by using the leaving-1/3-out approach. A randomized data set was divided into three parts, and 1/3 of samples were excluded to build a model with the remaining 2/3 of samples. Then, the excluded samples were predicted by the new model, and the process was repeated until all samples have been predicted at least once. Each time the percentage of correctly classified samples was calculated.

#### 3.5.7. Metabolite Identification

Compound ID—Putative identification of compound identity was performed by searching accurate masses against the online available databases as Kegg, Metlin, LipidMaps and HMDB using online available advanced CEU Mass Mediator tool. Isotopic distributions for each metabolite feature (LC-MS and CE-MS) have been studied for final confirmation. In-house developed CE-MS standards library was used for to compare relative migration time of selected metabolites. Compound identification by GC−MS was performed with the target metabolite Fiehn GC-MS Metabolomics RTL library (G1676AA, Agilent), the CEMBIO-library and the NIST mass spectra library 2008, using the ChemStation software and native PBM (Probability-Based Matching) algorithm (G1701EA E.02.00.493, Agilent).

### 3.6. Determination of Cholesterol and TAG in Liver

~60 mg of liver tissue was homogenized in 1 mL of PBS buffer using a homogenizer ultra-turrax^®^ (Ika) maintaining the sample tubes in ice. Then, 500 µL of liver homogenate were extracted with dichloromethane:methanol (2:1) and stirred for 1 h. Samples were centrifuged at 10,000 rpm for 5 min and pellets were re-extracted with dichloromethane. Lipid extracts were dried by using a Speed-Vac (Savant SPD131DDA-RVT4104TRAP, Thermo) and kept in −80 °C until analysis. Dry lipid samples were re-suspended in 250 μL of 2-propanol for the analysis. Total cholesterol (TC) and triacylglycerides (TAG) were determined in the liver extracts using a COBAS INTEGRA 400 plus system (Roche Diagnostics Ltd., Rotkreuz, Switzerland). Data were analyzed using one-way ANOVA. Homogeneity of variances was checked by Levene’s test. Tamhane’s T2 (equal variances not assumed) or Bonferroni (equal variances assumed) post hoc tests were used to determine differences within groups (*p* < 0.05). Analyses were performed using the IBM SPSS Statistics version 22 software (SPSS Inc., an IBM Company, Chicago, IL, USA).

## 4. Conclusions

The present study provides novel data about the hepatic metabolic signature of hypercholesterolemic Wistar rats and the potential action of an onion ingredient modulating the hypercholesterolemic pattern.

The comparison of the liver signatures among the three experimental groups indicated significant feature clusters in each technique. A total of 62 features for LC-MS, 33 features for CE-MS and 38 features for GC-MS were linked to potential metabolite candidates of group discrimination. The merging of these metabolites and the application of statistical tools provided a list of relevant metabolites that have an influential role in the separation of the HCO and HC group, which in turn signposts the metabolites strongly influenced by the consumption of the onion ingredient.

This broader picture revealed the potential pathways affected by HC and HCO diets, focusing the attention on disturbances of the HC group related to the lipid metabolism, the amino acid synthesis and metabolism, the carnitine and acylcarnitines homeostasis, the dysregulation of the TCA cycle and the formation of polyamines.

Hydroxybutyrylcarnitine and palmitoylcarnitine along with some glucosyl forms and methylated and free fatty acids were the main metabolites affected by the HCO group. Therefore, the energy and lipid metabolism including perturbations in the TCA cycle and β-oxidation, as well as the bile acid synthesis and possibly the methionine metabolism, reflected a significant impact in response to onion consumption at the core of hepatic dysfunctions.

However, the metabolites and pathways herein presented are preliminary and, although they may help generate new hypothesis about the preventive effects of onion consumption, further investigation to confirm the metabolite identities and additional validation of this model is required. Metabolomics hold potential to assess the effects of complementary dietetic approaches aimed to hepatic damage amelioration and NAFLD prevention.

## Figures and Tables

**Table 1:** List of variables selected by the combination of VIP-p(corr) (correlation coefficient combined with VIP, Variable Influence on the Projection) based on OPLS-DA model built for HCO vs. HC comparison. PC, phosphatidylcholine; PE, phosphoethanolamine; LPC, lysophosphatidylcholine; LPE, lysophosphatidylethanolamine; SM, sphingomyelin; DAG, diacylglycerol; TAG, triacylglycerol.

Compound Name	p(corr)	VIP	Compound Name	p(corr)	VIP
Hydroxybutyrylcarnitine	−0.66	2.5	Glycocholic acid	0.50	1.33
Glucopyranosyl-glucopyranosyl-glucose	0.55	2.26	PC(40:4) or PE(43:4)	0.69	1.32
Disaccharides (lactose)	0.60	2.23	LPC(32:0) or LPE(35:0)	−0.64	1.29
Palmitoylcarnitine	−0.70	2.13	Tricosanedioic acid	0.55	1.29
Methyl oleate	0.57	1.94	PC(33:3) or PE(36:3)	0.51	1.29
Methyl linolenate	0.78	1.86	PC(37:4) or PE(P-40:3)	0.69	1.29
Eicosadienoic acid	0.65	1.83	LPC(17:2) or LPE(20:2)	0.76	1.26
Cholenoic acid	0.70	1.83	PC(38:6)	0.65	1.26
Tetracosahexaenoic acid	0.72	1.78	PC(40:9)	0.50	1.25
Eicosenoic acid	0.69	1.75	Xanthine	−0.59	1.23
Methyl palmitate	0.82	1.71	PC(38:4) or PE(38:4)	−0.55	1.23
Docosatetraenoic acid	0.65	1.7	PC(37:4) or PE(40:4)	0.60	1.22
*O*-phosphocolamine	−0.63	1.7	Succinic acid	0.55	1.22
Oleic acid	0.73	1.67	DAG(42:8)	−0.56	1.22
Octadecatrienoic acid	0.79	1.63	SM(d44:1)	0.54	1.22
Glucose	0.56	1.59	Linolenoyl ethanolamide	0.65	1.21
Glycodeoxycholic acid	0.50	1.58	SM(33:1)	−0.57	1.2
Docosapentaenoic acid	0.64	1.57	PC(36:6)	0.50	1.19
Sedoheptulose	0.55	1.55	Heneicosatrienoic acid 21:3	0.58	1.15
Fumaric acid	−0.56	1.54	Docosahexaenoic acid methyl ester	0.74	1.13
Glucosylceramide (d34:1)	−0.57	1.54	Sugar alcohols C_6_H_14_O_6_	0.61	1.12
Glucosylceramide (34:0)	−0.57	1.54	PC(36:6) or PE(39:6)	0.50	1.11
Uracil	−0.59	1.52	PC(42:10)	0.55	1.11
LPC(O-13:1) or LPE(16:1)	0.71	1.49	PG(36:4)	−0.54	1.11
3-Hydroxybutyric acid	−0.58	1.47	PC(40:8)	0.50	1.11
Malic acid	−0.61	1.44	Dihydroxycholesterol	0.60	1.1
SM(d40:1)	−0.53	1.42	PE(38:7)	0.58	1.08
PC(P-38:4)	−0.62	1.4	Trans-hydroxy-proline	−0.59	1.08
Deoxyuridine	0.52	1.39	Tetrahydroxy-cholanoic acid	0.58	1.07
PC(36:5)	0.56	1.39	PC(40:6)	−0.65	1.05
Eicosatrienoic acid	0.55	1.38	PC(33:4) or PE(36:4)	0.52	1.04
Arachidonic acid	0.69	1.37	Dehydrosqualene	−0.61	1.03
TAG(54:8)	−0.58	1.35	PC(40:5)	0.61	1.01
Hexadecenoic acid	0.66	1.35	PC(38:2)	0.60	1.0

## Supplementary Materials

Supplementary materials can be found at www.mdpi.com/1422-0067/18/2/267/s1.
