# Supplementary material for: Metabolomic Fingerprinting in the Comprehensive Study of Liver Changes Associated with Onion Supplementation in Hypercholesterolemic Wistar Rats
Source: Int J Mol Sci. 2017 Jan 28;18(2):267. doi: 10.3390/ijms18020267 (PMC5343803; doi:10.3390/ijms18020267)
Supplement: Supplementary file 1 [file ijms-18-00267-s001.pdf]

Supplementary Materials: Metabolomic Fingerprinting in the Comprehensive Study of Liver Changes Associated with Onion Supplementation in Hypercholesterolemic Wistar Rats

Diana González-Peña, Danuta Dudzik, Antonia García, Begoña de Ancos, Coral Barbas and Concepción Sánchez-Moreno

Table S1. List of statistically significant metabolites identified by LC-MS.

Class Compound ID	Formula	RT	Mass	Error	CV	ESI	ANOVA	*p* Value and % Change		
								HC vs. C	HCO vs. HC	HCO vs. HC
Fatty Acyls										
Malic acid	$C_4H_6O_5$	0.81	134.0215	4	8.34	−	0.00600	NS<br>−2	0.00677<br>−42	0.00946<br>−41
Carnitine	$C_7H_{15}NO_3$	0.84	161.1052	4	2.55	+	0.00000	0.00000<br>−39	0.00000<br>−49	NS<br>−16
Dehydroxycarnitine	$C_7H_{15}NO_2$	1.09	145.1103	1	4.66	+	0.00419	0.00455<br>−59	0.00495<br>−59	NS<br>+1
Palmitoylcarnitine	$C_{23}H_{45}NO_4$	1.16	399.3349	2	2.09	+	0.03690	NS<br>−7	0.03083<br>−73	NS<br>−71
Oleoylcarnitine	$C_{25}H_{47}NO_4$	1.24	425.3505	1	2.81	+	0.02625	NS<br>−38	0.01264<br>−55	NS<br>−28
Hydroxyoctadecanoylcarnitine	$C_{25}H_{49}NO_5$	1.38	443.3611	0	4.94	+	0.00000	0.00000<br>+144	0.00000<br>+179	NS<br>+15
Hexadecenoic acid	$C_{16}H_{30}O_2$	1.87	254.2246	2	6.50	−	0.04563	NS<br>−49	NS<br>−17	NS<br>+61
Octadecatrienoic acid	$C_{18}H_{30}O_2$	2.48	278.2246	4	5.91	+	0.00849	NS<br>−14	0.03752<br>+38	0.00392<br>+60
Arachidonic Acid	$C_{20}H_{32}O_2$	2.03	304.2402	1	4.47	−	0.00010	0.00002<br>−59	0.00214<br>−40	NS<br>+47

Table S1. *Cont.*

Class Compound ID	Formula	RT	Mass	Error	CV	ESI	ANOVA	*p* Value and % Change		
								HC vs. C	HCO vs. HC	HCO vs. HC
Eicosatrienoic acid	$C_{20}H_{34}O_2$	2.39	306.2559	2	4.94	+	0.01290	NS<br>+155	0.00468<br>+291	NS<br>+53
Eicosadienoic acid	$C_{20}H_{36}O_2$	2.79	308.2715	8	11.07	−	0.03006	NS<br>+175	0.02094<br>+598	NS<br>+154
Eicosenoic acid	$C_{20}H_{38}O_2$	3.41	310.2872	7	5.70	−	0.00947	NS<br>+199	0.00444<br>+543	NS<br>+115
Docosapentaenoic acid	$C_{22}H_{34}O_2$	2.33	330.2559	5	7.49	−	0.03016	NS<br>+25	0.02461<br>+114	NS<br>+72
Tricosanedioic acid	$C_{23}H_{44}O_4$	2.79	384.3240	8	5.00	+	0.00866	NS<br>+156	0.00296<br>+281	NS<br>+49
Heneicosatrienoic acid 21:3	$C_{21}H_{36}O_2$	3.37	320.2715	4	4.76	+	0.00034	0.00498<br>+255	0.00008<br>+377	NS<br>+35
Docosatetraenoic acid	$C_{22}H_{36}O_2$	2.59	332.2715	7	4.95	−	0.01716	NS<br>+44	0.01195<br>+183	NS<br>+96
Docosatetraenoic acid	$C_{22}H_{36}O_2$	2.7	332.2715	0	1.81	+	0.01614	NS<br>+49	0.00696<br>+145	NS<br>+64
Tetracosahexaenoic acid	$C_{24}H_{36}O_2$	2.47	356.2715	7	4.27	−	0.00662	NS<br>+124	0.00320<br>+397	0.04896<br>+122
Docosahexaenoic acid methyl ester	$C_{23}H_{34}O_2$	2.78	342.2559	3	3.49	+	0.00001	0.00000<br>−51	0.00017<br>−36	NS<br>+30
Methyl stearate	$C_{19}H_{38}O_2$	4.75	298.2872	4	15.04	+	0.00000	0.00000<br>−59	0.00000<br>−54	NS<br>+12
Amines										
Linolenoyl ethanolamide or Anandamide (18:3, n-6)	$C_{20}H_{35}NO_2$	2.11	321.2668	0	4.33	+	0.00061	0.00017<br>−53	0.00695<br>−37	NS<br>+35

Table S1. *Cont.*

Class Compound ID	Formula	RT	Mass	Error	CV	ESI	ANOVA	*p* Value and % Change		
								HC vs. C	HCO vs. HC	HCO vs. HC
Steroids and steroid derivatives										
Androsterone or Stearidonic acid methyl ester	$C_{19}H_{30}O_2$	2.07	290.2246	1	7.24	+	0.00944	NS −10	0.01923 +64	0.00660 +81
Methyl linoleate	$C_{19}H_{32}O_2$	2.47	292.2402	2	4.32	+	0.01405	NS −5	0.02302 +47	0.01145 +55
Tetrahydroxy-cholanoic acid	$C_{24}H_{40}O_6$	1.67	424.2825	8	7.88	−	0.00035	0.00012 -53	0.00362 -39	NS +32
Norcholestanol	$C_{26}H_{46}O$	2.35	374.3549	1	2.32	+	0.00015	0.00007 −72	0.00040 −62	NS +35
Dihydroxycholesterol	$C_{27}H_{46}O_3$	2.62	418.3447	1	3.25	+	0.00001	0.00039 +594	0.00000 +961	0.02629 +53
Cholenoic acid	$C_{24}H_{38}O_2$	2.94	358.2872	11	4.77	−	0.02419	NS +67	0.01847 +292	NS +135
Hydroxycholanoic acid	$C_{39}H_{50}O_3$	3	566.3760	11	6.70	+	0.00000	0.00000 −66	0.00000 −68	NS −5
Ketocholesterol	$C_{27}H_{44}O_2$	4.55	400.3341	1	8.18	+	0.00000	0.00005 +550	0.00000 +745	NS +30
Dehydrocholesterol	$C_{27}H_{44}O$	23.9	384.3392	2	10.32	+	0.00000	0.00000 +1643	0.00000 +1706	NS +4
Cholesteryl eicosapentaenoate	$C_{47}H_{74}O_2$	27.14	670.5689	10	16.60	+	0.00000	0.00000 +567	0.00000 +706	NS +21
Cholesterol	$C_{27}H_{46}O$	27.66	386.3549	1	16.16	+	0.00000	0.00000 HC	0.00000 CO	NS +9
20:1-Glc-cholesterol	$C_{53}H_{92}O_7$	27.77	840.6843	3	16.30	+	0.00000	0.00000 +680	0.00000 +713	NS +4
Cholesteryl palmitoleate	$C_{43}H_{74}O_2$	28.27	622.5689	0	6.16	+	0.00000	0.00000 +1290	0.00000 +1394	NS +8

Table S1. *Cont.*

Class Compound ID	Formula	RT	Mass	Error	CV	ESI	ANOVA	*p* Value and % Change		
								HC vs. C	HCO vs. HC	HCO vs. HC
22:2-Glc-cholesterol	$C_{55}H_{94}O_7$	28.36	866.7000	4	19.51	+	0.00000	0.00000<br>+213	0.00000<br>+248	NS<br>+11
Cholesteryl oleate	$C_{45}H_{78}O_2$	30.34	650.6002	1	18.79	+	0.00000	0.00000<br>+613	0.00000<br>+666	NS<br>+8
22:1-Glc-Campesterol	$C_{56}H_{96}O_6$	30.35	882.7313	1	7.32	+	0.00000	0.00000<br>+889	0.00000<br>+1024	NS<br>+14
Glycocholic acid	$C_{26}H_{43}NO_6$	0.86	465.3091	2	5.86	−	0.00035	0.00458<br>+4437	0.00011<br>+6353	NS<br>+42
Glycodeoxycholic acid	$C_{26}H_{43}NO_5$	0.87	449.3141	2	3.22	−	0.00745	0.03760<br>+2598	0.00414<br>+3505	NS<br>+34
Lineolic acids and derivatives										
9-OxoODE	$C_{18}H_{30}O_3$	1.61	294.2195	5	8.98	−	0.00028	0.00465<br>+98	0.00008<br>+144	NS<br>+23
Carbohydrates and carbohydrate conjugates										
Gulose 1-phosphate	$C_6H_{13}O_9P$	0.95	260.0297	2	4.36	−	0.00000	0.00000<br>−58	0.00000<br>−48	NS<br>+23
Glucopyranosyl-glucopyranosyl-glucose	$C_{18}H_{32}O_{16}$	0.76	504.1690	2	2.77	+	0.01217	0.01374<br>−59	NS<br>0	0.01376<br>+143
Glucose	$C_6H_{12}O_6$	0.75	180.0634	4	6.11	−	0.00368	0.00228<br>−39	NS<br>−7	0.01315<br>+52
Sedoheptulose	$C_7H_{14}O_7$	0.77	210.0739	1	4.78	−	0.00142	0.00048<br>−45	NS<br>−16	0.02062<br>+54
Lactose	$C_{12}H_{22}O_{11}$	0.8	342.1162	6	2.51	−	0.00321	NS<br>−34	NS<br>+45	0.00111<br>+121
Mevalonic acid 5-phosphate	$C_6H_{13}O_7P$	0.91	228.0399	3	7.23	−	0.01791	NS<br>+171	0.01198<br>+232	NS<br>+23

Table S1. *Cont.*

Class Compound ID	Formula	RT	Mass	Error	CV	ESI	ANOVA	*p* Value and % Change		
								HC vs. C	HCO vs. HC	HCO vs. HC
Prenol lipids										
Retinol	$C_{20}H_{30}O$	2.87	286.2297	1	6.38	+	0.00000	0.00000<br>−48	0.00001<br>−37	NS<br>+22
Dehydrosqualene	$C_{30}H_{48}$	6.6	408.3756	2	3.21	+	0.00017	0.00003<br>+122	0.06187<br>+53	0.01106<br>−31
Trimethyl-tridecanoic acid	$C_{16}H_{32}O_2$	2.27	256.2402	0	5.97	−	0.00000	0.00000<br>-64	0.00000<br>−58	NS<br>+16
Alkaloids and derivatives										
Uric acid	$C_5H_4N_4O_3$	0.81	168.0283	1	3.75	−	0.00220	0.00129<br>+233	0.00812<br>+190	NS<br>−13
Xanthine	$C_5H_4N_4O_2$	0.81	152.0334	9	5.70	−	0.00809	NS<br>+9	0.03067<br>−32	0.00523<br>−37
Phosphate										
Glycerophosphoglycerol	$C_6H_{15}O_8P$	0.88	246.0504	1	7.63	−	0.00001	0.00001<br>−47	0.00000<br>−50	NS<br>−6
Sphingolipids										
Glucosylceramide (34:0)	$C_{40}H_{77}NO_8$	7.12	699.5649	3	3.74	−	0.00196	0.00073<br>+211	NS<br>+69	0.02208<br>−46
Glucosylceramide (d34:1)	$C_{40}H_{77}NO_8$	7.71	699.5649	4	3.18	+	0.00137	0.00039<br>+233	NS<br>+79	0.01699<br>−46
Glucosylceramide (42:1)	$C_{48}H_{93}NO_8$	16.49	811.6901	2	19.64	−	0.00100	0.00037<br>+104	0.01078<br>+73	NS<br>−15
Lactosylceramide (d18:1/16:0)	$C_{46}H_{87}NO_{13}$	6.63	861.6177	2	2.51	−	0.00176	0.00052<br>+124	NS<br>+60	NS<br>−29
SM(33:1)	$C_{38}H_{77}N_2O_6P$	7.35	688.5519	3	1.95	−	0.01439	NS<br>+35	NS<br>−10	0.01021<br>-34

Table S1. *Cont.*

Class Compound ID	Formula	RT	Mass	Error	CV	ESI	ANOVA	*p* Value and % Change		
								HC vs. C	HCO vs. HC	HCO vs. HC
SM(32:1)	$C_{37}H_{75}N_2O_6P$	6.25	674.5363	5	3.00	+	0.00000	0.00000<br>-44	0.00000<br>-50	NS<br>-11
SM(d40:1)	$C_{45}H_{91}N_2O_6P$	15.47	786.6615	1	5.51	+	0.00101	0.00024<br>+272	NS<br>+118	0.03425<br>−42
SM(d44:1)	$C_{49}H_{97}N2O_6P$	18.81	840.7084	3	6.09	+	0.00000	0.00014<br>+7099	0.00000<br>+10532	NS<br>+48
Glycerophospholipids										
PC(30:2) or PE(33:2)	$C_{38}H_{72}NO_8P$	7.88	701.4996	2	0.60	−	0.00000	0.00000<br>+160	0.00000<br>+147	NS<br>−5
PC(32:2) or PE(35:2)	$C_{40}H_{76}NO_8P$	6.87	729.5309	0	2.47	−	0.00050	0.00442<br>+79	0.00019<br>+108	NS<br>+16
PC(32:2) or PE(35:2)	$C_{40}H_{76}NO_8P$	7.51	729.5309	1	2.22	+	0.00024	0.00178<br>+85	0.00007<br>+114	NS<br>+16
PC(33:1) or PE(36:1)	$C_{41}H_{80}NO_8P$	12.76	745.5621	9	14.84	−	0.00010	0.00002<br>+238	0.00866<br>+134	0.04863<br>−31
PC(33:1) or PE(36:1)	$C_{41}H_{80}NO_8P$	9.93	745.5621	3	6.72	+	0.00000	0.00000<br>+225	0.00000<br>+249	NS<br>+7
PC(33:2)	$C_{41}H_{78}NO_8P$	8.5	743.5465	2	4.53	+	0.00000	0.00000<br>+264	0.00000<br>+278	NS<br>+4
PC(33:2) or PE(36:2)	$C_{41}H_{78}NO_8P$	11.17	743.5465	2	12.99	−	0.00000	0.00000<br>+182	0.00000<br>+162	NS<br>-7
PC(33:3) or PE(36:3)	$C_{41}H_{76}NO_8P$	10.71	741.5309	7	2.80	−	0.00000	0.00000<br>+237	0.00005<br>+158	NS<br>−24
PC(33:3) or PE(36:3)	$C_{41}H_{76}NO_8P$	7.6	741.5309	0	5.83	+	0.00013	0.04920<br>+74	0.00002<br>+168	0.00992<br>+54
PC(33:4) or PE(36:4)	$C_{41}H_{74}NO_8P$	8.32	739.5152	1	5.60	−	0.00000	0.00000<br>+193	0.00000<br>+199	NS<br>+2

Table S1. *Cont.*

Class Compound ID	Formula	RT	Mass	Error	CV	ESI	ANOVA	*p* Value and % Change		
								HC vs. C	HCO vs. HC	HCO vs. HC
PC(33:4) or PE(36:4)	$C_{41}H_{74}NO_8P$	8.93	739.5152	1	4.86	+	0.00002	0.00015<br>+167	0.00001<br>+214	NS<br>+18
PC(33:5) or PE(36:5)	$C_{41}H_{72}NO_8P$	8.27	737.4996	2	4.53	+	0.01896	NS<br>−14	NS<br>+30	0.00871<br>+51
PC(34:3) or PE(37:3)	$C_{42}H_{78}NO_8P$	8.22	755.5465	3	3.29	+	0.00005	0.00145<br>+133	0.00001<br>+203	NS<br>+30
PC(35:2) or PE(38:2)	$C_{43}H_{82}NO_8P$	13.65	771.5778	10	9.76	−	0.00000	0.00000<br>+161	0.00000<br>+154	NS<br>−3
PC(35:3) or PE(38:3)	$C_{43}H_{80}NO_8P$	8.46	769.5621	3	5.80	−	0.00000	0.00000<br>+234	0.00000<br>+248	NS<br>+4
PC(35:3) or PE(38:3)	$C_{43}H_{80}NO_8P$	9.24	769.5621	2	7.81	+	0.00000	0.00000<br>+320	0.00000<br>+412	NS<br>+22
PC(36:2)	$C_{44}H8_4NO_8P$	11.84	785.5934	2	3.85	+	0.00000	0.00000<br>+113	0.00000<br>+125	NS<br>+6
PC(36:3) or PE(39:3)	$C_{44}H_{82}NO_8P$	9.44	783.5778	7	12.81	−	0.00000	0.00000<br>+113	0.00000<br>+132	NS<br>+9
PC(36:5)	$C_{44}H_{78}NO_8P$	7.84	779.5465	0	11.07	+	0.00230	NS<br>+53	0.00061<br>+119	NS<br>+43
PC(36:4)	$C_{44}H_{80}NO_8P$	8.83	781.5621	2	3.01	+	0.00000	0.00001<br>+155	0.00000<br>+198	NS<br>+17
PC(36:6)	$C_{44}H_{76}NO_8P$	7.38	777.5309	0	3.32	+	0.00004	0.00001<br>−58	0.00116<br>−38	NS<br>+48
PC(36:6) or PE(39:6)	$C_{44}H_{76}NO_8P$	6.78	777.5309	3	6.64	−	0.00015	0.00003<br>−50	0.00698<br>−30	NS<br>+40
PC(37:2) or PE(40:2)	$C_{45}H_{86}NO_8P$	12.18	799.6091	3	15.11	−	0.00005	0.00226<br>+69	0.00001<br>+110	NS<br>+24

Table S1. *Cont.*

Class Compound ID	Formula	RT	Mass	Error	CV	ESI	ANOVA	*p* Value and % Change		
								HC vs. C	HCO vs. HC	HCO vs. HC
PC(37:3) or PE(40:3)	$C_{45}H_{84}NO_8P$	10.56	797.5934	6	18.11	−	0.00000	0.00000<br>+189	0.00000<br>+208	NS<br>+7
PC(37:4) or PE(P-40:3)	$C_{45}H_{84}NO_7P$	2.01	781.5986	6	6.95	−	0.00001	0.00000<br>−68	0.00005<br>−54	NS<br>+40
PC(37:4) or PE(40:4)	$C_{45}H_{82}NO_8P$	10.79	795.5778	1	3.83	+	0.00000	0.00000<br>−63	0.00000<br>−52	NS<br>+30
PC(37:5) or PE(40:5)	$C_{45}H_{80}NO_8P$	12.5	793.5621	4	14.22	−	0.00006	0.00008<br>−51	0.00006<br>−52	NS<br>−3
PC(38:2) or PE(41:2)	$C_{46}H_{88}NO_8P$	13.68	813.6248	0	12.86	−	0.00016	0.04165<br>+49	0.00003<br>+105	0.01664<br>+38
PC(38:2)	$C_{46}H_{88}NO_8P$	14.77	813.6248	1	7.01	+	0.00000	0.00013<br>+88	0.00000<br>+137	0.02492<br>+27
PC(38:3) or PE(41:3)	$C_{46}H_{86}NO_8P$	11.99	811.6091	2	16.07	−	0.00000	0.00031<br>+74	0.00000<br>+136	0.00230<br>+35
PC(38:4) or PE(38:4)	$C_{46}H_{84}NO_7P$	10.88	793.5985	3	14.97	−	0.00006	0.00001<br>+346	0.01134<br>+181	0.02201<br>−37
PC(P-38:4)	$C_{46}H_{84}NO_7P$	11.76	793.5985	6	10.83	+	0.00059	0.00012<br>+230	NS<br>+113	0.04709<br>−36
PC(38:6)	$C_{46}H_{80}NO_8P$	8.22	805.5621	1	4.92	−	0.00000	0.00000<br>−56	0.00001<br>−41	NS<br>+34
PC(38:6)	$C_{46}H_{80}NO_8P$	9.3	805.5621	2	4.88	+	0.00000	0.00000<br>−63	0.00000<br>−58	NS<br>+13
PC(38:7)	$C_{46}H_{78}NO_8P$	9.3	803.5465	2	2.69	+	0.00000	0.00000<br>−59	0.00000<br>−53	NS<br>+15
PC(40:4) or PE(43:4)	$C_{48}H_{88}NO_8P$	13.75	837.6248	0	8.51	−	0.00075	0.00021<br>−46	NS<br>-18	0.02128<br>+52

Table S1. *Cont.*

Class Compound ID	Formula	RT	Mass	Error	CV	ESI	ANOVA	*p* Value and % Change		
								HC vs. C	HCO vs. HC	HCO vs. HC
PC(40:5)	$C_{48}H_{86}NO_8P$	11.86	835.6091	7	13.90	−	0.00035	0.00011<br>−53	0.00421<br>−37	NS<br>+33
PC(40:5)	$C_{48}H_{86}NO_8P$	13.24	835.6091	6	5.26	+	0.00144	0.00069<br>−53	0.00467<br>−44	NS<br>+21
PC(40:6)	$C_{48}H_{84}NO_8P$	11.73	833.5934	2	2.15	+	0.00003	0.00000<br>+108	0.00783<br>+54	0.00913<br>−26
PC(40:7)	$C_{48}H_{82}NO_8P$	12.44	831.5778	2	8.35	+	0.00010	0.00007<br>+105	0.00014<br>+100	NS<br>−3
PC(40:8)	$C_{48}H_{80}NO_8P$	8.72	829.5621	0	2.10	+	0.00000	0.00000<br>−78	0.00001<br>−68	NS<br>+43
PC(40:9)	$C_{48}H_{78}NO_8P$	7.62	827.5465	1	6.80	+	0.00001	0.00001<br>−72	0.00002<br>−69	NS<br>+9
PC(42:10)	$C_{50}H_{80}NO_8P$	7.91	853.5621	2	2.27	−	0.00041	0.00033<br>-85	0.00083<br>−78	NS<br>+46
PC(42:10)	$C_{50}H_{80}NO_8P$	8.61	853.5621	2	2.37	+	0.00090	0.00064<br>−85	0.00157<br>−78	NS<br>+48
PE(38:7)	$C_{43}H_{72}NO_8P$	8.12	761.4996	2	4.79	+	0.00383	NS<br>−24	NS<br>+15	0.00122<br>+51
PE(P-38:5)	$C_{43}H_{76}NO_7P$	11.71	749.5359	1	7.63	+	0.00001	0.00000<br>+189	0.00001<br>+180	NS<br>−3
PE(36:2)	$C_{41}H_{78}NO_8P$	12.05	743.5465	2	5.30	+	0.00000	0.00000<br>+305	0.00000<br>+226	0.01071<br>−19
PE(42:5)	$C_{47}H_{84}NO_8P$	11.52	821.5934	2	11.37	+	0.00000	0.00000<br>+134	0.00000<br>+109	0.07553<br>−11
LPC(O-13:1) or LPE(16:1)	$C_{21}H_{42}NO_7P$	1.26	451.2699	8	4.72	−	0.00115	NS<br>+39	0.00045<br>+136	0.01021<br>+70

Table S1. *Cont.*

Class Compound ID	Formula	RT	Mass	Error	CV	ESI	ANOVA	*p* Value and % Change		
								HC vs. C	HCO vs. HC	HCO vs. HC
LPC(14:0) or LPE(17:0)	$C_{22}H_{46}NO_7P$	1.67	467.3012	7	3.90	−	0.00263	0.00041<br>+100	0.00017<br>+130	NS<br>+15
LPC(16:1) or LPE(19:1)	$C_{24}H_{48}NO_7P$	1.27	493.3168	11	4.05	−	0.00032	0.02346<br>+68	0.00007<br>+124	0.06987<br>+34
LPC(17:0) or LPE(20:0)	$C_{25}H_{52}NO_7P$	2.58	509.3481	2	5.51	−	0.00035	0.00103<br>+107	0.00021<br>+123	NS<br>+8
LPC(17:2) or LPE(20:2)	$C_{25}H_{48}NO_7P$	1.73	505.3168	11	1.55	−	0.00191	NS<br>+56	0.00057<br>+116	NS<br>+39
LPC(32:0) or LPE(35:0)	$C_{40}H_{80}NO_{8P}$	9.36	733.5621	9	5.12	−	0.02166	NS<br>+12	NS<br>-26	0.01377<br>−34
LPC(33:0) or LPE(36:0)	$C_{41}H_{84}NO_7P$	2.24	733.5986	1	9.87	−	0.00000	0.00000<br>−66	0.00000<br>−62	NS<br>+12
LPC(18:2)	$C_{26}H_{50}NO_7P$	1.39	519.3325	0	1.61	−	0.00000	0.00000<br>+104	0.00000<br>+98	NS<br>−3
LPC(20:1)	$C_{28}H_{56}NO_7P$	2.13	549.3795	2	2.51	−	0.00001	0.00051<br>+75	0.00000<br>+112	NS<br>+21
LPC(20:4)	$C_{28}H_{50}NO_7P$	1.38	543.3325	0	1.55	−	0.00000	0.00000<br>−50	0.00000<br>−46	NS<br>+7
LPC(22:6)	$C_{30}H_{50}NO_7P$	1.32	567.3325	0	2.23	−	0.00000	0.00000<br>−65	0.00000<br>−61	NS<br>+11
LPC(31:0) or LPE(34:0)	$C_{39}H_{78}NO_8P$	9.17	719.5465	2	7.29	+	0.00000	0.00000<br>+133	0.00002<br>+87	0.01482<br>−20
LPC(32:0) or LPE(35:0)	$C_{40}H_{80}NO_8P$	10.14	733.5621	2	1.99	+	0.01981	NS<br>+14	NS<br>−25	0.00838<br>−34
PC(40:9)	$C_{48}H_{78}NO_8P$	7.05	827.5465	4	3.82	−	0.00003	0.00001<br>−72	0.00019<br>−58	NS<br>+49

Table S1. *Cont.*

Class Compound ID	Formula	RT	Mass	Error	CV	ESI	ANOVA	p Value and % Change		
								HC vs. C	HCO vs. HC	HCO vs. HC
PG(36:4)	$C_{42}H_{75}O_{10}P$	7.28	770.5098	1	6.16	−	0.00001	0.00000<br>+648	0.00086<br>+381	NS<br>−36
Glycerolipids										
DAG(34:2)	$C_{37}H_{68}O_5$	13.21	592.5067	1	5.65	+	0.00002	0.00015<br>+83	0.00001<br>+107	NS<br>+13
DAG (36:2)	$C_{39}H_{72}O_5$	9.96	620.5380	1	12.49	+	0.00001	0.00001<br>+207	0.00001<br>+211	NS<br>+2
DAG (36:3)	$C_{39}H_{70}O_5$	14.1	618.5223	2	10.01	+	0.00000	0.00000<br>+183	0.00000<br>+254	0.02124<br>+25
DAG (36:5)	$C_{39}H_{66}O_5$	10.76	614.4910	0	4.01	+	0.00000	0.00000<br>+142	0.00000<br>+164	NS<br>+9
DAG (38:3)	$C_{41}H_{74}O_5$	16.59	646.5536	1	3.50	+	0.00053	0.00028<br>+127	0.00133<br>+110	NS<br>−8
DAG (38:4)	$C_{41}H_{72}O_5$	10.45	644.5380	1	4.35	+	0.00000	0.00000<br>+288	0.00000<br>+284	NS<br>−1
DAG (38:5)	$C_{41}H_{70}O_5$	13.34	642.5223	1	10.85	+	0.00044	0.00010<br>+526	0.00842<br>+339	NS<br>−30
DAG (38:6)	$C_{41}H_{68}O_5$	12.32	640.5067	2	5.21	+	0.00003	0.00005<br>+151	0.00002<br>+162	NS<br>+4
DAG (40:6)	$C_{43}H_{72}O_5$	15.25	668.5380	4	5.50	+	0.00001	0.00000<br>+138	0.00005<br>+112	NS<br>−11
TAG (37:5)	$C_{54}H_{94}O_6$	28.17	838.7050	10	6.14	+	0.00000	0.00001<br>−65	0.00000<br>−71	NS<br>−17
DAG (40:7)	$C_{43}H_{70}O_5$	15.04	666.5223	4	4.36	+	0.00002	0.00000<br>+184	0.00031<br>+134	NS<br>−18
DAG (40:8)	$C_{43}H_{68}O_5$	12.09	664.5067	2	3.73	+	0.00000	0.00000<br>+219	0.00000<br>+169	0.01430<br>−16

Table S1. *Cont.*

Class Compound ID	Formula	RT	Mass	Error	CV	ESI	ANOVA	*p* Value and % Change		
								HC vs. C	HCO vs. HC	HCO vs. HC
DAG (42:8)	$C_{45}H_{72}O_5$	14.38	692.5380	11	3.06	+	0.00209	0.00063<br>+111	NS<br>+38	0.02390<br>−34
TAG (45:0)	$C_{48}H_{92}O_6$	28.6	764.6894	1	16.69	+	0.00000	0.00000<br>−73	0.00000<br>−76	NS<br>−11
TAG (46:1)	$C_{49}H_{92}O_6$	28.42	776.6894	0	16.27	+	0.00042	0.00090<br>−54	0.00024<br>−61	NS<br>−15
TAG (48:3)	$C_{51}H_{92}O_6$	27.78	817.7288	15	9.61	+	0.02940	NS<br>−45	0.02260<br>−51	NS<br>−11
TAG (50:2)	$C_{53}H_{98}O_6$	30.62	830.7363	3	18.51	+	0.00009	0.00008<br>−74	0.00009<br>−73	NS<br>+4
TAG (50:4)	$C_{53}H_{94}O_6$	28.38	826.7050	0	19.66	+	0.00050	0.00085<br>−63	0.00034<br>−68	NS<br>−15
TAG (50:3n6)	$C_{52}H_{94}O_6$	28.66	814.7050	7	8.73	+	0.00000	0.00001<br>−54	0.00000<br>−61	NS<br>−16
TAG (51:4n6)	$C_{54}H_{96}O_6$	29.28	840.7207	18	11.01	+	0.00000	0.00000<br>−65	0.00000<br>−69	NS<br>−12
TAG (52:5)	$C_{55}H_{96}O_6$	29.13	852.7207	13	8.52	+	0.00000	0.00000<br>−70	0.00000<br>−72	NS<br>−8
TAG (52:6)	$C_{55}H_{94}O_6$	28.03	850.7050	10	10.60	+	0.00003	0.00005<br>−59	0.00001<br>−65	NS<br>−15
TAG (52:7n6)	$C_{55}H_{92}O_6$	27.19	843.7319	10	18.61	+	0.00068	0.00100<br>−48	0.00056<br>−51	NS<br>−5

Table S1. *Cont.*

Class Compound ID	Formula	RT	Mass	Error	CV	ESI	ANOVA	*p* Value and % Change		
								HC vs. C	HCO vs. HC	HCO vs. HC
TAG (54:7)	$C_{57}H_{96}O_6$	28.6	876.7207	2	13.43	+	0.00000	0.00000<br>−70	0.00001<br>−65	NS<br>+19
TAG (54:8)	$C_{57}H_{94}O_6$	27.5	874.7050	17	9.41	+	0.00030	0.00108<br>−51	0.00012<br>−63	NS<br>−23

C—Control group; HC—High-cholesterol fed group; HCO— High-cholesterol enriched with onion fed group; % change represents the increase (+) or decrease (−) of the mean in the specified, the sign indicates the direction of the change. $p < 0.05$ was considered significant. *p* values corrected by Benjamini-Hochberg (FDR, false discovery rate) and Bonferroni test, respectively. NS—data not significant. Mass—Monoisotopic molecular weight; Error—mass error in PPM; RT—Retention time; CV–Coefficient of variation calculated for quality control; LPC—lysophosphatidylcholine; LPE—lysophosphatidylethanolamine; PC—phosphatidylcholine; PE—phosphoethanolamine; SM—sphingomyelin; DAG—diacylglycerol; TAG—triacylglycerol.

Table S2. List of statistically significant metabolites identified by CE-MS.

Class Compound ID	Formula	MT	Mass	Error	CV	ANOVA	*p* Value and % Change		
							HC vs. C	HCO vs. C	HCO vs. HC
Carboxylic acids and derivatives									
**Serine**	$C_3H_7NO_3$	19.85	105.0426	3	3.91	0.00066	0.00092<br>−23	0.00055<br>−24	NS<br>−1
**Valine**	$C_5H_{11}NO_2$	22.57	117.079	1	3.64	0.00273	0.00070<br>+72	NS<br>+38	NS<br>−20
**Histidine**	$C_6H_9N_3O_2$	14.72	155.0695	3	4.06	0.00000	0.00000<br>−31	0.00002<br>−23	NS<br>+13
**Glycine**	$C_2H_5NO_2$	16.52	75.0320	12	3.72	0.00017	0.00625<br>−17	0.00003<br>−28	NS<br>−14
**Methylhistidine**	$C_7H_{11}N_3O_2$	15.13	169.0851	5	5.67	0.00041	NS<br>−20	0.00008<br>−42	NS<br>−27
**Pipecolic acid**	$C_6H_{11}NO_2$	13.89	129.079	1	4.60	0.00000	0.00000<br>−35	0.00000<br>−38	NS<br>−6

Table S2. *Cont.*

Class Compound ID	Formula	MT	Mass	Error	CV	ANOVA	*p* Value and % Change		
							HC vs. C	HCO vs. C	HCO vs. HC
Organic acids and derivatives									
**Aspartic acid**	$C_4H_7NO_4$	22.87	133.0375	3	4.36	0.00027	0.01041<br>−32	0.00005<br>−53	NS<br>−31
**Asparagine**	$C_4H_8N_2O_3$	20.82	132.0535	4	3.73	0.00593	0.00007<br>22	NS<br>13	0.00122<br>−8
**Oxidized glutathione**	$C_{20}H_{32}N_6O_{12}S_2$	24.64	612.1520	0	3.04	0.00000	0.00000<br>−46	0.00000<br>−42	NS<br>+8
Dipeptides									
**Val-Ala**	$C_8H_{16}N_2O_3$	19.95	188.1161	1	4.65	0.00962	0.00837<br>−27	0.01397<br>−25	NS<br>+2
**Ile- Ser**	$C_9H_{18}N_2O_4$	20.88	218.1267	3	3.80	0.00849	0.01086<br>−26	0.00793<br>−27	NS<br>−1
**Val-Leu**	$C_{11}H_{22}N_2O_3$	21.31	230.1630	1	4.19	0.00117	0.00465<br>−25	0.00049<br>−32	NS<br>−9
**Thr-Leu**	$C_{10}H_{20}N_2O_4$	21.32	232.1423	2	4.80	0.01230	0.02631<br>−28	0.00817<br>−32	NS<br>−7
**Leu-Ile**	$C_{12}H_{24}N_2O_3$	21.73	244.1787	3	3.80	0.01554	0.04972<br>−20	0.00860<br>−25	NS<br>−7
**Thr-Phe**	$C_{13}H_{18}N_2O_4$	21.61	266.1267	5	5.03	0.00962	0.00554<br>−27	0.02547<br>−22	NS<br>+7
**Leu-Phe**	$C_{15}H_{22}N_2O_3$	21.97	278.1630	4	5.17	0.00009	0.00017<br>−32	0.00005<br>−35	NS<br>−5
**Leu-Arg**	$C_{12}H_{25}N_5O_3$	15.16	287.1957	1	4.93	0.00001	0.00001<br>−47	0.00000<br>−50	NS<br>−5

Table S2. *Cont.*

Class Compound ID	Formula	MT	Mass	Error	CV	ANOVA	*p* Value and % Change		
							HC vs. C	HCO vs. C	HCO vs. HC
Fatty Acyls									
**Threonine**	$C_4H_9NO_3$	20.94	119.0582	3	4.86	0.00215	0.00553<br>−21	0.00111<br>−26	NS<br>−5
**Carnitine**	$C_7H_{15}NO_3$	17.2	161.1052	2	4.31	0.00001	0.00083<br>−25	0.00000<br>−40	0.03586<br>−21
**Dehydroxycarnitine**	$C_7H_{15}NO_2$	17.56	145.1103	3	5.60	0.00043	0.00027<br>−43	0.00083<br>−39	NS<br>+7
**Hydroxybutyrylcarnitine**	$C_{11}H_{21}NO_5$	19.91	247.1420	20	8.37	0.00022	0.00007<br>+1963	NS<br>+440	0.00122<br>−74
Imidazolines									
**Creatinine**	$C_4H_7N_3O$	14.66	113.0589	0	12.81	0.02941	NS<br>−3	0.02157<br>−17	NS<br>−15
Organonitrogen compounds									
**Spermidine**	$C_7H_{19}N_3$	9.17	145.1579	0	3.09	0.00001	0.00042<br>−22	0.00000<br>−36	0.02820<br>−18
Amines									
**Spermine**	$C_{10}H_{26}N_4$	9.13	202.2157	1	5.92	0.00000	0.00000<br>−27	0.00000<br>−29	NS<br>−3
Purines and purine derivatives									
**Adenine**	$C_5H_5N_5$	15.53	135.0545	3	18.66	0.00000	0.00000<br>−62	0.00000<br>−56	NS<br>+13
**Hypoxanthine**	$C_5H_4N_4O$	24.39	136.0385	4	4.53	0.00188	NS<br>−7	0.00061<br>−23	0.01247<br>−18
**Adenosine**	$C_{10}H_{13}N_5O_4$	20.26	267.0968	1	3.80	0.00001	0.00000<br>−53	0.00004<br>−45	NS<br>+17

Table S2. *Cont.*

Class Compound ID	Formula	MT	Mass	Error	CV	ANOVA	*p* Value and % Change		
							HC vs. C	HCO vs. C	HCO vs. HC
Pyridines and derivatives									
**Dimethyluracil**	$C_6H_8N_2O_2$	16.42	140.0586	2	11.86	0.02202	0.02103<br>+94	0.02910<br>+89	NS<br>−2
**Methylnicotinamide**	$C_7H_8N_2O$	14.69	136.0637	13	2.48	0.00001	0.00001<br>−65	0.00001<br>−63	NS<br>+5
Carbohydrates and carbohydrate conjugates									
***S*-Adenosyl-homocysteine**	$C_{14}H_{20}N_6O_5S$	17.86	384.1216	4	4.70	0.00000	0.00000<br>−48	0.00000<br>−44	NS<br>+7
***S*-Adenosyl-methionine**	$C_{15}H_{22}N_6O_5S$	14.65	398.1372	2	2.47	0.00027	0.00009<br>−47	0.00139<br>−37	NS<br>+19
**Choline**	$C_5H_{13}NO$	13.65	103.0997	2	5.46	0.00027	0.00005<br>+79	0.01036<br>+47	NS<br>−18
**Hydroxymethylthio butanoic acid**	$C_5H_{10}O_3S$	17.93	150.0351	4	9.78	0.02684	0.02907<br>−13	0.03150<br>−13	NS<br>0

C—Control group; HC—High-cholesterol fed group; HCO— High-cholesterol enriched with onion fed group; % change represents the increase (+) or decrease (−) of the mean in the specified, the sign indicates the direction of the change. p <0.05 was considered significant. p values corrected by Benjamini-Hochberg (FDR, false discovery rate) and Bonferroni test, respectively. NS—data not significant. Mass—Monoisotopic molecular weight; MT—Migration time; Error—mass error in PPM; CV—Coefficient of variation calculated for quality control.

Table S3. List of statistically significant metabolites identified by GC-MS.

Class Compound ID	Formula	RT	Mass	CV	ANOVA	*p* Value and % Change		
						HC vs. C	HCO vs. C	HCO vs. HC
Organic acids and derivatives								
**Alanine**	$C_3H_7NO_2$	7.45	89.04768	5	0.00039	0.00033<br>−27	0.00032<br>−27	NS<br>0
**Serine**	$C_3H_7NO_3$	11.06	105.0426	3	0.00039	0.00114<br>−23	0.00012<br>−28	NS<br>−7
**Lysine**	$C_6H_{14}N_2O_2$	17.63	146.1055	5	0.00000	0.00000<br>−41	0.00000<br>−45	NS<br>−7
**Glycine**	$C_2H_5NO_2$	10.34	75.03203	8	0.00001	0.00004<br>−36	0.00000<br>−48	NS<br>−19
**Aspartic acid**	$C_4H_7NO_4$	13.12	133.0375	17	0.00553	0.04668<br>-36	0.00147<br>-56	NS<br>-32
**Trans-hydroxy-proline**	$C_5H_9NO_3$	13.20	131.0582	8	0.04490	NS<br>−12	0.02379<br>−42	NS<br>−34
**Succinic acid**	$C_4H_6O_4$	10.43	118.0266	6	0.00006	0.00279<br>+188	0.00001<br>+317	0.04379<br>+45
**Fumaric acid**	$C_4H_4O_4$	10.92	116.011	10	0.00089	NS<br>−22	0.00019<br>−65	0.01051<br>−55
**Aminomalonic acid**	$C_3H_5NO_4$	12.50	119.0219	20	0.00319	0.01373<br>−26	0.00103<br>−35	NS<br>−12
**Urea**	CH_4N_2O	9.46	60.0323	6	0.00004	0.00004<br>−30	0.00001<br>−33	NS<br>−4

Table S3. *Cont.*

Class Compound ID	Formula	RT	Mass	CV	ANOVA	*p* Value and % Change		
						HC vs. C	HCO vs. C	HCO vs. HC
				Fatty Acyls				
**Threonine**	$C_4H_9NO_3$	11.40	119.0582	9	0.00290	0.03439<br>−19	0.00067<br>−31	NS<br>−15
**Malic acid**	$C_4H_6O_5$	12.71	134.0215	5	0.00011	0.04245<br>−27	0.00001<br>−62	0.00602<br>−49
**2-Hydroxybutyric acid**	$C_4H_8O_3$	7.79	104.0473	9	0.03950	NS<br>−21	0.02389<br>−45	NS<br>−31
**3-Hydroxybutyric acid**	$C_4H_8O_3$	8.28	104.0473	10	0.03032	NS<br>+1	0.03371<br>-53	0.03142<br>-53
**Arachidonic acid**	$C_{20}H_{32}O_2$	21.76	304.2402	23	0.03137	0.01699<br>−33	NS<br>−8	NS<br>+36
**Palmitic acid**	$C_{16}H_{32}O_2$	18.86	256.2402	10	0.00736	0.00199<br>−38	0.06703<br>−24	NS<br>+24
**Oleic acid**	$C_{18}H_{34}O_2$	20.44	282.2559	5	0.01623	NS<br>+30	0.00869<br>+150	0.04054<br>+92
**Methyl linolenate**	$C_{19}H_{34}O_2$	19.35	294.2559	12	0.01344	NS<br>−5	0.01529<br>+103	0.01100<br>+113
**Methyl oleate**	$C_{19}H_{36}O_2$	19.42	296.2720	21	0.02272	NS<br>−40	NS<br>+63	0.00887<br>+173
**Methyl stearate**	$C_{19}H_{38}O_2$	19.65	298.2872	11	0.00000	0.00000<br>−51	0.00000<br>−50	NS<br>+2
**Methyl palmitate**	$C_{17}H_{34}O_2$	17.70	270.2559	14	0.00034	0.00004<br>−61	NS<br>−26	0.01249<br>+91
**Tyrosine**	$C_9H_{11}NO_3$	17.79	181.0739	1	0.04178	0.04417<br>−17	NS<br>−14	NS<br>+3

Table S3. *Cont.*

Class Compound ID	Formula	RT	Mass	CV	ANOVA	*p* Value and % Change		
						HC vs. C	HCO vs. C	HCO vs. HC
Phenylpropanoic acids								
**Tryptophan**	$C_{11}H_{12}N_2O_2$	20.42	204.0899	25	0.00285	0.00356<br>−8	0.00168<br>−52	NS<br>−8
Indoles and derivatives								
**Dehydroascorbic acid**	$C_6H_6O_6$	16.84	174.0164	18	0.01344	0.00395<br>−40	NS<br>−21	NS<br>+30
**Uric acid**	$C_5H_4N_4O_3$	19.31	168.0283	6	0.01372	0.02282<br>+171	0.00888<br>+195	NS<br>+9
Diazines								
**Uracil**	$C_4H_4N_2O_2$	10.75	112.0273	8	0.00015	0.00002<br>+501	0.02305<br>+205	0.00569<br>−49
Purines and purine derivatives								
**Hypoxanthine**	$C_5H_4N_4O$	16.42	136.0385	7	0.01372	NS<br>−7	0.00544<br>−24	NS<br>−18
**Inosine**	$C_{10}H_{12}N_4O_5$	23.36	268.0808	4	0.00000	0.00000<br>−45	0.00000<br>−40	NS<br>+9
**Adenosine**	$C_{10}H_{13}N_5O_4$	23.85	267.0968	9	0.00001	0.00000<br>−68	0.00001<br>−61	NS<br>+23
Pyridines and derivatives								
**Nicotinamide**	$C_6H_6N_2O$	12.70	122.0480	12	0.00000	0.00000<br>−46	0.00000<br>−41	NS<br>+10
**Deoxyuridine**	$C_9H_{12}N_2O_5$	22.36	228.0746	21	0.00248	0.00076<br>−55	0.00850<br>−43	NS<br>+29

Table S3. *Cont.*

Class Compound ID	Formula	RT	Mass	CV	ANOVA	*p* Value and % Change		
						HC vs. C	HCO vs. C	HCO vs. HC
Non-metal oxoanionic compounds								
**Pyrophosphate**	O_7P_2	14.80	173.9119	15	0.00000	0.00000<br>−40	0.00000<br>−36	NS<br>+7
***O*-phosphocolamine**	$C_2H_8NO_4P$	16.16	141.0191	20	0.00049	0.00010<br>+361	NS<br>+111	0.00470<br>−54
Steroids and steroid derivatives								
**Cholesterol**	$C_{27}H_{46}O$	27.58	386.3549	7	0.00006	0.00001<br>+74	0.00029<br>+57	NS<br>−10
Carbohydrates and carbohydate conjugates								
**Glucose-6-phosphate**	$C_6H_{13}O_9P$	21.30	260.0297	7	0.00000	0.00000<br>−81	0.00000<br>−70	NS<br>+56
**Disaccharides**	$C_{12}H_22O_{11}$	24.82	342.1162	6	0.00755	NS<br>−46	0.04961<br>+23	0.00230<br>+129
**Sugar alcohols**	$C_4H_{10}O_4$	12.98	122.0579	12	0.02283	0.03459<br>−36	0.01823<br>−40	NS<br>−6
**Sugar alcohols**	$C_6H_{14}O_6$	18.03	182.0790	28	0.00039	0.00011<br>−66	0.00172<br>−51	NS<br>+44

C—Control group; HC—High-cholesterol fed group; HCO— High-cholesterol enriched with onion fed group; % change represents the increase (+) or decrease (−) of the mean in the specified, the sign indicates the direction of the change. $p < 0.05$ was considered significant. *p* values corrected by Benjamini-Hochberg (FDR, false discovery rate) and Bonferroni test, respectively. NS—data not significant. Mass—Monoisotopic molecular weight; RT—Retention time; Error—mass error in PPM; CV—Coefficient of variation calculated for quality control.

Table S4. Composition of the experimental diets [control (C), high-cholesterol (HC) and high-cholesterol enriched with onion (HCO)].

Ingredient (g/kg)	C	HC	HCO
Onion powder	–	–	100
Casein	200	200	200
Sucrose	100	100	100
Maize starch	470.49	445.49	368.69
Soya oil	50	50	50
Maize oil	80	80	80
Mineral mixture *	35	35	35
Vitamin mixture †	10	10	10
Cellulose powder	50	50	26.8
Choline bitartrate	2.5	2.5	2.5
tert-butylhydroquinone	0.010	0.010	0.010
L-cystine	2	2	2
Cholesterol	–	20	20
Cholic acid	–	5	5

* Mineral mix for the AIN-93M diet, g/kg (AIN-93M-MX): calcium carbonate anhydrous, 357.00; potassium phosphate monobasic, 250.00; potassium citrate, tripotassium monohydrate, 28.00; sodium chloride, 74.00; potassium sulphate, 46.00; magnesium oxide, 24.00; ferric citrate, 6.06; zinc carbonate, 1.65; sodium meta-silicate $9H_2O$, 1.45; manganous carbonate, 0.63; cupric carbonate, 0.30; chromium potassium sulfate $12H_2O$, 0.275; boric acid, 0.0815; sodium fluoride, 0.0635; nickel carbonate, 0.0318; lithium chloride, 0.0174; sodium selenate anhydrous, 0.01025; potassium iodate, 0.0100; ammonium paramolybdate $4H_2O$, 0.00795; ammonium vanadate, 0.0066; powdered sucrose, 209.806. † Vitamin mix for the AIN-93M diet, g/kg (AIN-93-VX): niacin, 3.000; calcium pantothenate, 1.600; pyridoxine-HCl, 0.700; thiamin-HCl, 0.600; riboflavin, 0.600; folic acid, 0.200; biotin, 0.200; vitamin B12 (0.1%), 2.500; vitamin E (all-*rac*-α-tocopheryl acetate, 500 IU/g), 15.000; vitamin A (all-*trans*-retinyl palmitate, 500,000 IU/g), 0.800; vitamin D3 (400,000 IU/g), 0.250; vitamin K1, 0.075; powdered sucrose, 974.655. ‡ Diet energy content was calculated using the factors 16.73 kJ/g (4 kcal/g) for protein, 15.69 kJ/g (3.75 kcal/g) for monosaccharides, 16.53 kJ/g (3.95 kcal/g) for disaccharides, 17.49 kJ/g (4.18 kcal/g) for starch, 8.37 kJ/g (2 kcal/g) for dietary fibre, and 37.65 kJ/g for fat. Control diet, 18540.9 kJ/kg (4431.4 kcal/kg); HC diet, 18856.6 kJ/kg (4506.8 kcal/kg); HCO diet, 18642.4 kJ/kg (4455.6 kcal/kg).

Table S5. Nutritional composition, phytochemical compounds, and antioxidant activity of onion powder.

Onion Powder	Mean ± SD
Protein (g/100 g)	9.75 ± 0.08
Lipids (g/100 g)	1.30 ± 0.06
Carbohydrates (g/100 g)	80.10 ± 2.89
Glucose (g/100 g)	27.7 ± 1.73
Fructose (g/100 g)	20.7 ± 0.46
Sucrose (g/100 g)	4.3 ± 0.11
Total fructans (g/100 g)	4.2 ± 0.10
Total dietary fibre (g/100 g)	23.2 ± 1.15
Soluble fibre (g/100 g)	3.2 ± 0.09
Insoluble fibre (g/100 g)	20.0 ± 0.17
Ash (g/100 g)	4.63 ± 0.07
Total phenols (mg GAE/100 g)	1629.6 ± 60.0
Quercetin 3-glucoside (mg/100 g)	32.22 ± 0.90
Quercetin 4′-glucoside (mg/100 g)	950.00 ± 2.99
Quercetin 3,4′-diglucoside (mg/100 g)	1368.89 ± 8.77

Table S5. *Cont.*

Onion Powder	Mean ± SD
Quercetin 7,4′-diglucoside (mg/100 g)	31.56 ± 0.33
Quercetin 3,7,4′-triglucoside (mg/100 g)	9.16 ± 0.32
Isorhamnetin 4′-glucoside (mg/100 g)	45.16 ± 1.54
Isorhamnetin 3,4′-diglucoside (mg/100 g)	32.00 ± 0.19
Total ACSOs (mg BCSOE/100 g)	4120.89 ± 89.43
Propionaldehyde (mg/100 g)	245.04 ± 39.61
1-Propanethiol (mg/100 g)	23.54 ± 0.90
Hexanal (mg/100 g)	0.04 ± 0.001
2-Methyl 2-pentenal (mg/100 g)	10.80 ± 0.67
Propyl thioacetate (mg/100 g)	0.45 ± 0.03
Dimethyl trisulfide (mg/100 g)	66.41 ± 5.02
Dipropyl disulfide (mg/100 g)	89.45 ± 3.29
Methyl propyl trisulfide (mg/100 g)	42.28 ± 2.14
Dipropyl trisulfide (mg/100 g)	25.50 ± 2.45
Ascorbic acid (mg/100 g)	62.31 ± 0.77
Total vitamin C (mg/100 g)	104.26 ± 4.07
Scavenging of $NO^{•}$ (μmol TE/100 g)	1706.00 ± 49.61
$ABTS^{•+}$ (μmol TE/100 g)	4936.67 ± 72.65
$DPPH^{•}$ (μmol TE/100 g)	1135.00 ± 82.21
FRAP (μmol TE/100 g)	12245.14 ± 60.45

Values are expressed as the mean ± SD (*n* = 3). GAE, gallic acid equivalents; ACSOs, *S*-alk(en)yl-L-cysteine sulfoxide; BCSOE, *S*-Butyl-L-cysteine sulfoxide equivalents; $NO^{•}$, nitric oxide radical $ABTS^{•+}$, 2,2′-azinobis(3-ethylbenzothiazoline-6-sulfonic acid) radical cation; $DPPH^{•}$, 2,2-diphenyl-1-picrylhydrazyl radical; FRAP, ferric reducing antioxidant power; TE, trolox equivalents.

Onion Ingredient Preparation

The onion ingredient was prepared by using raw onions (*Allium cepa* L. var *cepa*, "Recas") harvested in Spain, supplied by Cebacat (Asociación Catalana de Productores-Comercializadores de Cebolla). Onions free from external damages (stored at 4 °C until processing) were hand-peeled, cut into 10 mm diced-pieces, packaged in bags with very low gas permeability (Doypack®, Polyskin XL, Amcor Flexibles Hispania, S.L., Granollers, Barcelona, Spain) and treated with high pressure. The high-pressure treatment (400 MPa, 5 min, 25 °C) was applied in a High Pressure Iso-Lab System (Model FPG7100:9/2C, Stansted Fluid Power Ltd., Essex, UK).The high-pressurised diced onion was immediately frozen with liquid nitrogen, freezedried in a lyophiliser (model Lyoalfa, Telstar, S.A., Barcelona, Spain), and pulverised with an ultra centrifugal mill ZM 200 (Retsch GmbH, Haan, Germany), obtaining a fine powder with a size particle of ≤250 μm, which was stored at −20 ± 0.5 °C until use.
